# Identification of miRNAs Contributing to the Broad-Spectrum and Durable Blast Resistance in the Yunnan Local Rice Germplasm

**DOI:** 10.3389/fpls.2021.749919

**Published:** 2021-10-14

**Authors:** Jinlu Li, Hui Zhang, Rui Yang, Qianchun Zeng, Guangyu Han, Yunlong Du, Jing Yang, Genhua Yang, Qiong Luo

**Affiliations:** ^1^State Key Laboratory for Conservation and Utilization of Bio-Resources in Yunnan/Ministry of Education Key Laboratory of Agricultural Biodiversity for Plant Disease Management, Yunnan Agricultural University, Kunming, China; ^2^College of Agronomy and Biotechnology, Yunnan Agricultural University, Kunming, China

**Keywords:** rice, miRNA, Ziyu44, *M. oryzae*, miR9664, *R* genes

## Abstract

MicroRNAs are 20–24 nucleotide non-coding RNAs and play important roles in plant-environment interactions. In recent years, many microRNAs (miRNAs) have been found to regulate rice immunity against rice blast fungus. However, there are limited studies about miRNAs that directly target resistance (*R*) genes to regulate rice immunity. In this study, by deep sequencing, small RNA libraries were constructed from four-leaf stage seedlings of the resistant variety Ziyu44 and susceptible variety Jiangnanxiangnuo (JNXN) upon *Magnaporthe oryzae* infection, we found that much more miRNAs were significantly differentially expressed in Ziyu44 than in JNXN. Among these miRNAs, we focused on miR9664, a newly identified rice miRNA in our sequencing, which was upregulated lightly in Ziyu44 and drastically in JNXN at 24–48 h post-inoculation (hpi). The transgenic plants overexpressing miR9664 (miR9664-oe) displayed reduced defense responses to *M. oryzae*, while those knocking down miR9664 (miR9664-m) displayed enhanced defense responses to *M. oryzae*. Most of the detected miR9664 predicted target genes were reduced in the miR9664-oe lines while increased in the miR9664-m lines. The cleavage site of *LOC_Os08g07774* was confirmed by RLM-RACE. Meanwhile, after being inoculated with *M. oryzae*, the genes were expressed differently between Ziyu44 and JNXN. The results suggest that miR9664-mediated *R* gene turnover contributes to Ziyu44 broad-spectrum resistance to rice blast fungus. Taken together, our research identified a new rice miRNA that directly targets *R* genes to regulate rice immunity against rice blast fungus, adding significant information to the study of rice–*M. oryzae* interaction.

## Introduction

Rice is a staple food for more than half of the population of the world, hence, rice blast, caused by the fungal pathogen *Magnaporthe oryzae*, severely threatens global food security and sustainable agriculture (Zhou, 2016). The development of broad-spectrum and durable-resistance varieties is considered the most economical and eco-friendly strategy to control this disease (Dangl et al., 2013). In the past decades, there has been significant progress in the identification of broad-spectrum resistance genes and in the understanding of the molecular basis of broad-spectrum resistance to blast disease (Li W. et al., 2019, 2020). However, in comparison with *indica* rice, the research on broad-spectrum resistance mechanisms in *japonica* rice is rather limited due to the lack of resistance resources (Shang et al., 2009). There remain many gaps in our understanding of the effects of broad-spectrum resistance to rice blast disease (Li W. et al., 2019), and there are still many challenges regarding the application of broad-spectrum resistance in crop breeding (Li W. et al., 2020).

Recently, an increasing number of studies have shown that microRNAs (miRNAs) are involved in plant immunity (Baldrich and San Segundo, 2016). miRNAs are 20–24 nucleotide endogenous non-coding RNAs abundant in eukaryotes, and negatively regulate the gene expression by the mRNA cleavage, translational repression, and DNA methylation, which is based on sequence complementarity (Huang et al., 2019; Song et al., 2019). Once attacked by pathogenic microbes, host plants can recognize conserved pathogen-associated molecular patterns (PAMPs) and activate PAMP-triggered immunity (PTI) (Zipfel, 2008), which is the basal defense in plants. In *Arabidopsis*, the PAMP molecule, flg22, can induce miR393 which promotes basal defense against the virulent *Pseudomonas syringae* DC3000 by silencing the F-box auxin receptors to suppress auxin signaling (Navarro et al., 2006). Pathogens subvert PTI by delivering various effector proteins into plant cells. In turn, these effectors could be recognized by resistance (*R*) proteins in plants, leading to a stronger effector-triggered immunity (ETI), which is often associated with hypersensitive response (HR) (Jones and Dangl, 2006; Liu and Wang, 2016). In barley, the miR398-*CSD1* module can contribute to an *R*-gene *mildew resistance locus A (MLA)*-mediated ETI. Upon being attacked by barley powdery mildew fungus, the activation of *MLA* can downregulate miR398 to derepress its target gene *CSD1*, thereby enhancing *CSD1*-mediated reactive oxygen species (ROS) accumulation and cell death (Xu et al., 2014).

In the past decade, a panel of miRNAs have been identified to target various *R* genes in plant genomes (Baldrich and San Segundo, 2016; Huang et al., 2019). miRNAs reduce *R* gene levels under normal conditions and allow the induction of *R* gene expression under various stresses (Deng et al., 2018). The 22-nt miRNAs, miR2118, miR1507, and miR2109, were initially found to target nucleotide-binding and leucine-rich repeat (*NB-LRR*) genes (the largest group of plant *R* genes) and trigger the production of phased secondary small interfering RNAs (phasiRNAs) in *Medicago* (Zhai et al., 2011). Then, miR6019 and miR6020 targeting the *TIR-NB-LRR immune receptor N* gene were identified in tobacco (Li et al., 2012). In tomatoes, miR482, miR2118, and miR5300 targeting *NB-LRRs* were identified (Shivaprasad et al., 2012; Ouyang et al., 2014). miR9863 targeting *MLA* alleles to attenuate NOD-like receptor (NLR)-triggered disease resistance was identified in barley (Liu et al., 2014). Md-miRln20 negatively regulates the resistance of apples to Glomerella leaf spot (GLS) (caused by *Glomerella cingulata*) by suppressing Md-TN1-GLS (contains a Toll-like/interleukin-1 receptor domain and an NBS) expression (Zhang Y. et al., 2019). Lineage-specific evolved miRNAs regulate different types of *NBS-LRR* genes in *Triticeae* to act in response to biotic and abiotic stresses (Zhang R. et al., 2019). Several 22-nt miRNAs were identified to target *R* genes and trigger the production of 21-nt phased sRNAs (phasiRNAs) after being infected with *potato virus Y* (Prigigallo et al., 2019). miR1885 targets both the TIR-NBS-LRR class of *R* gene *BraTNL1* and photosynthesis-related gene *BraCP24* for negative regulation through distinct modes of action (Cui et al., 2020). To this date, about 70 *R* gene-targeting miRNAs have been identified in different plant genomes (Deng et al., 2018; Prigigallo et al., 2019; Zhang R. et al., 2019; Zhang Y. et al., 2019; Cui et al., 2020).

Increasing pieces of evidence indicate that miRNAs play important roles in the regulation of rice blast interactions by effectively and accurately regulating the expression of their target genes. The overexpression of miR160a enhanced the resistance of rice to rice blast disease (Li et al., 2014). To boost rice immunity against the blast fungus *M. oryzae*, osa-miR398b targeted multiple superoxide dismutase genes (Li Y. et al., 2019). osa-miR7695 repressed an alternatively spliced transcript of *OsNramp6* (*Natural resistance-associated macrophage protein6*), resulting in an enhanced rice immunity to *M. oryzae* (Campo et al., 2013). Both miR166k and miR166h positively regulated rice immunity to *M. oryzae via* post-transcriptional control of ethylene insensitive 2 (EIN2) (Salvador-Guirao et al., 2018). miR169a negatively regulated rice immunity against the blast fungus by repressing the expression of the nuclear factor *Y-A* (*NF-YA*) gene (Li Y. et al., 2017). osa-miR167d negatively regulated the immunity of rice to *M. oryzae* by downregulating auxin response factor 12 *(ARF12*) (Zhao et al., 2019). miR396 negatively regulated rice blast disease resistance *via* suppressing multiple *OsGRFs* (*growth-regulating factors*) (Chandran et al., 2019). miR164a targeted *OsNAC60* and negatively regulated rice immunity against rice blast fungus (Wang Z. et al., 2018). miR319b repressed the expression of *TEOSINTE BRANCHED /CYCLOIDEA/PROLIFERATING CELL FACTOR1* (*OsTCP21*) and *LIPOXYGENASE2 (LOX2)/LOX5*(a key enzyme of JA synthesis), negatively regulating the rice immunity against the blast fungus *M. oryzae* (Zhang et al., 2018). miR444b.2 negatively regulated rice immunity against *M. oryzae via* suppressing the tiller-determinant MADS-box family genes (Xiao et al., 2016). osa-miR1873-targeted *LOC_Os05g01790* to negatively regulate rice immunity against *M. oryzae* (Zhou et al., 2020). osa-miR162 targets *OsDCL1* (*Dicer-like 1*) to fine-tune the immunity of rice against *M. oryzae* and yield traits (Li X. P. et al., 2020).

Ziyu44 (*Oryza sativa* L. subsp. *geng*), a local variety in Yunnan, a province of China, displayed broad-spectrum resistance to 16 physiological races, namely, ZA1, ZA49, ZA57, ZA61; ZB1, ZB13, ZB17, ZB25; ZC1, ZC3, ZC13, ZC15; ZE1, ZE3; ZF1, and ZG (Zhang et al., 2011). Over the past 30 years, Ziyu44 has also displayed highly durable field blast resistance in several blast endemic areas, including Yiliang of Yunnan province, Yangzhou of Jiangsu province, Enshi of Hubei province, and Lingshui of Hainan province (Zhuo et al., 2019). In our previous study, we identified multiple major and minor resistance loci in Ziyu44 and suggested that the combination of major and minor resistance on multiple loci is one of the mechanisms underlying the durable resistance of Ziyu44 to rice blast disease (Zhang et al., 2009; Zhou et al., 2015; Hu et al., 2017; Zhuo et al., 2019). However, the molecular mechanisms underlying the durable and broad-spectrum resistance to rice blast disease in Ziyu44 are largely unknown.

In this study, to further explore the broad-spectrum resistance mechanisms in Ziyu44 from the perspective of the miRNAs, we carried out an identification of the miRNAs contributing to the broad-spectrum and durable blast resistance in Ziyu44 through deep sequencing small RNA libraries constructed from four-leaf stage seedlings of the resistant variety Ziyu44 and susceptible variety JNXN upon *M. oryzae* infection. Further, we performed a functional investigation of miR9664, a novel miRNA identified in our study, by generating the overexpression and knockdown transgenic lines in the rice variety, Taipei309 (TP309), background. Our data indicate that miRNAs play important roles in the immunity of Ziyu44 against *M. oryzae* infection, and lay an important foundation for the identification of new genes for rice blast resistance and uncovering the molecular basis of broad-spectrum resistance to the blast disease in Ziyu44.

## Materials and Methods

### Plant Materials and Growth Conditions

The rice (*Oryza sativa*) blast-resistance *japonica* variety Ziyu44, and blast-susceptible *japonica* varieties JNXN, TP309, and miR9664 transgenic lines were grown in a growth room maintained at 26°C and 70% relative humidity with a cycle of 12 h light and 12 h dark period. *Arabidopsis thaliana* (*Col-0*) was grown in a greenhouse at 24°C with a 16 h light period.

### Fungal Materials and Growth Conditions

Twelve *M. oryzae* strains were used in this study. TC61, LP11, LP33, YZ121, JS5, ZB13, ZB15, HN2, and H63 were isolated from the Yunnan, Jiangsu, Sichuan, Hainan, and Heilongjiang provinces of China, respectively, and were stored in our lab. 81278 and Zhong-10-8-14 were kindly provided by Prof. Lihuang Zhu (State Key Laboratory of Plant Genomics, Institute of Genetics and Developmental Biology, Chinese Academy of Sciences). Prof. Mo Wang, from the Fujian University Key Laboratory for Plant-Microbe Interaction, Fujian Agriculture and Forestry University, kindly provided Guy11. First, *M. oryzae* was cultured in a Potato Dextrose Agar (PDA) at 28°C under 12 h light/12 h darkness for ~3 days, then, picked mycelia at the edge of the colony were cultured in a Potato Dextrose Broth (PDB) at 28°C, 180 rpm for ~4 days, and finally, 700 μl of mycelium solution were spread on the tomato oat media, at 28°C under 12 h light/12 h darkness for ~5 days.

### Pathogen Infection Assay and Disease Evaluation

For the spraying inoculation, the spores of *M. oryzae* strains were collected from the tomato oat media and adjusted to a concentration of 1–2 × 10^5^ spore ml^−1^. The spores of each strain were mixed with the inoculum and sprayed onto the four-leaf-stage seedlings of rice. Afterward, the materials were incubated with moisture and returned to normal light conditions after 24 h of shading. The disease symptoms were recorded at 5 days post-inoculation (dpi), and the lesion types were assessed from 0 (resistance) to 5 (susceptible).

For the punching inoculation, about 6 cm fragments of rice leaves were cut from the same part at tillering stage, and two evenly spaced wounds were punctured on the leaves using a needle. The leaves were then soaked in water containing 1 mg/L 6-BA at pH 7, and 10 μl of spore suspension (1 × 10^5^ spores ml^−1^) was dropped on the leaf wound sites. The material was then shaded for 24 h, and then returned to normal light conditions. The disease lesions were pictured 6 days after the inoculation, and the lesion length was measured using a caliper.

For the leaf sheath-inoculation, one of the tillering leaf sheaths of rice was peeled off, and the spore suspension (0.5 × 10^5^ spore ml^−1^) was slowly injected into the leaf sheaths, then cultivated in a dark and moisturizing manner. After 48 h of inoculation, the epidermis of the leaf sheath was cut off with a razor blade to observe the growth of spores.

### Library Construction, Sequencing, and Bioinformatics Analysis of Small RNA-Sequencing

The spores of 10 *M. oryzae* strains were collected and adjusted to a concentration of 2 × 10^5^ spore ml^−1^. The spores of each strain were mixed with the inoculum and sprayed onto the Ziyu44 and JNXN four-leaf-stage seedlings. Based on the *M. oryzae* infection cycle (Ribot et al., 2008; Wilson and Talbot, 2009), the leaves treated with *M. oryzae* at 0, 3, 10, and 22 hpi were collected and were immediately frozen in liquid nitrogen before storing at −80°C until use. This was performed in three independent experiments under similar conditions. The total RNA from the 24 samples was extracted using Trizol, and the RNA molecules with the size range of 18–30 nt were enriched with polyacrylamide gel electrophoresis (PAGE). Then, 3′adapters were added and the 36–44 nt RNAs were enriched. Afterward, 5′adapters were ligated to the RNAs. The ligation products were reverse transcribed through PCR amplification and the 140~160 bp sized PCR products were enriched to generate a complementary DNA (cDNA) library; the libraries were sequenced using the Illumina HiSeq^TM^ 2500 by Gene Denovo Biotechnology Co. (Guangzhou, China). The raw reads obtained were filtered according to the following rules: (1) low quality reads containing more than one low quality (*Q* ≤ 20) base or containing unknown nucleotides (N) were removed; (2) reads without 3′adapters, reads containing 5′adapters, and reads containing 3′ and 5′ adapters but no small RNA fragments between them, were removed; (3) reads containing polyA in small RNA fragments were removed; (4) reads shorter than 18 nt were removed. Further, the clean reads were aligned with the small RNAs in the GeneBank database (Release 209.0) and Rfam database (11.0) to identify the ribosomal RNA (rRNA), small nuclear RNA (snRNA), small nucleolar RNA (snoRNA), and transfer ribonucleic acid (tRNA). The clean reads were also aligned with the reference genome to identify small RNA sequences mapped to the exons, introns, or repeat sequences. The clean reads were then searched against the miRBase database (Release 21) to identify the osa-miRNA and known miRNA (miRNAs alignment with other species). The unannotated reads were used to predict the novel miRNAs using Mireap_v0.2 (Mireap software is a perl script program that needs to be used with VienaRNA1.7, which written by Ivo Hofacker). Then based on the expression of the total miRNAs (osa-miRNA, known miRNA, and novel miRNA), the miRNA expression level was calculated and normalized to transcripts per million (TPM). The analysis of the significantly differentially expressed miRNAs and target gene prediction were described in detail in the main part of the article.

### RNA Isolation and RT-qPCR

The total RNA was extracted using a TaKaRa MiniBEST Plant RNA Extraction Kit (TaKaRa, Dalian, China) according to the protocols of the manufacturer. The RNA concentration and quality were determined using a NanoDrop 2000 UVevis Spectrophotometer (Thermo Fisher Scientific, Waltham, MA, USA) and the integrity distribution was examined by electrophoresis in 2% agarose gel. The first strain cDNAs were synthesized using a ReverTra Ace qPCR RT Master Mix with a gDNA Remover kit (TOYOBO, Shanghai, China) for the expression level analysis of predicted target genes. To check the miRNA expression, we used the stem-loop method to design the primer and a PrimeScript^TM^ RT reagent Kit (Takara, Japan) to reverse the transcription of miRNAs (Chen et al., 2005). The reverse-transcription PCR (RT-qPCR) was conducted using the Bio-Rad CFX96 Real-Time System coupled with a C1000 Thermal Cycler (Bio-Rad, Hercules, CA, USA). The reference gene, Ubiquitin, was used for the normalization of the predicted target genes from the RT-qPCR results, and U6 snRNA was used for the normalization of miRNAs RT-qPCR results. The 2^−Δ*ΔCT*^ method was used to calculate the relative expression level with three-technique repeats. The primers used in this study are listed in Supplementary Table 5.

### Construction of Transgenic Plants

To construct the miR9664 over-expression plasmid, first, we replaced the miR319a in the *MIR319a* (AT4G23713) sequence with miR9664 by overlapping the PCR through three steps (Schwab et al., 2006): step 1, a genomic fragment of *MIR319a* (AT4G23713) was amplified from *Arabidopsis* Col-0 by PCR using the primer pairs *MIR319*-F and *MIR319*-R; step 2, three independent PCR amplifications were performed using the products of the PCR from step 1 as templates with *MIR319-F*/IV-miR9664, II-miR9664/III-miR9664, andI-miR9664/*MIR319*-R primer pairs; step 3, PCR amplification was performed using the mixture of the three independent PCR amplification products from step 2 as templates with *MIR319*-F/*MIR319*-R primer pairs. Afterward, the PCR products obtained in step 3 were joined to vector using the homologous recombination strategy (Seamless Assembly Cloning Kit, Beijing, China), and the colonies containing the correct sequence were selected and stored. To construct the miR9664 knock-down plasmid (over-expressing target mimicry of miR9664), the artificial target mimicry sequences of osa-miR9664 were inserted into the *IPS1* gene to substitute for the miR399 target site with the primers *IPS1*-F, miR9664mimic-F, miR9664mimic-R, and *IPS1*-R as previously described (Wang et al., 2015) and were cloned into the *BamH* I site of the vector pCAMBIA1300 which was kindly provided by Prof. Zhukuan Cheng from the State Key Laboratory of Plant Genomics, Institute of Genetics and Developmental Biology, Chinese Academy of Sciences, resulting in the over-expressing target mimicry of the miR9664 plasmid. Both constructs were transformed into TP309 via an *Agrobacterium* strain EHA105-mediated transformation. Hygromycin B was used to screen the positive transgenic lines. The primers used in this study are listed in Supplementary Table 5.

### 5′RLM-RACE Assay

The 5′ RNA ligation-mediated rapid amplification of cDNA ends (5′RLM-RACE) assay was performed using a FirstChoice RLM-RACE kit (Amion, Part Number AM1700) according to the protocol of the manufacturer. Three micrograms of the total RNA extracted from miR9664-oe#2 were used for the 5′RACE adapter ligation. Afterward, we proceeded to assemble the reverse transcription reaction, outer 5′RLM-RACE PCR, and inner 5′RLM-RACE PCR. Finally, the PCR products were cloned using pEASY-Blunt Cloning Vector (TransGen), and sequence analysis was conducted on the products. The primers used in this study are listed in Supplementary Table 5.

## Results

### Deep Sequencing of Small RNA Libraries

The four-leaf-stage seedlings of Ziyu44 and JNXN were inoculated with a conidial mixture of 10 *M. oryzae* strains isolated from different rice-growing areas in China. Infected leaves were collected at 3, 10, and 22 hpi, and leaves before the inoculation were used as a control (0 hpi). Three independent experiments under similar conditions were conducted. Twenty-four small RNA libraries were constructed and subjected to sequencing. All clean reads are shown in Supplementary Table 1.

**Figure 1:**
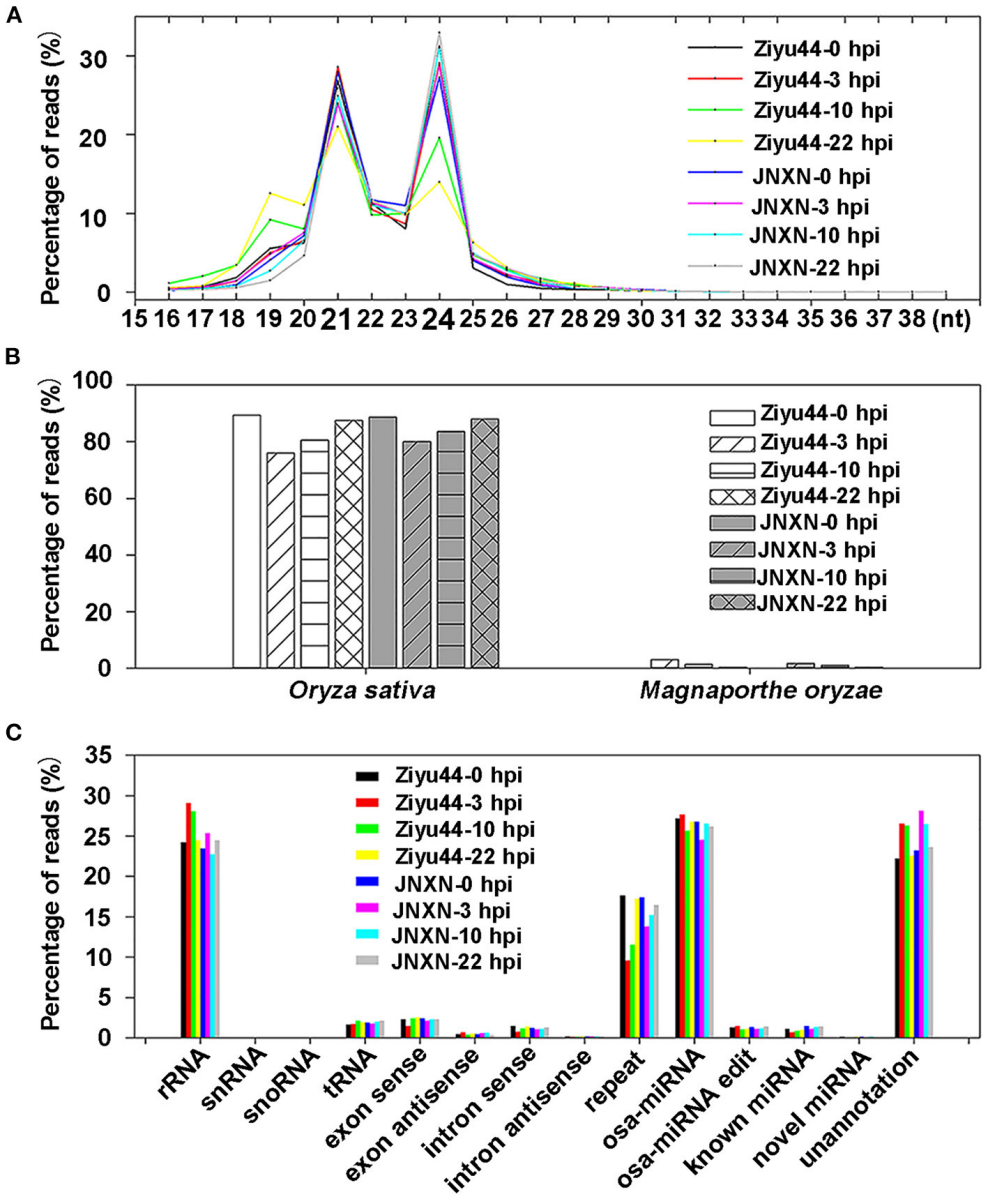
Analysis of small RNA sequencing data. **(A)** Size distribution of small RNAs reads. **(B)** The bar charts show the percentage of small RNA reads matched to the rice and *Magnaporthe oryzae* genomes. **(C)** The bar charts show the percentage of small RNA reads matched to the different rice RNAs commentary in the GeneBank/Rfam database, reference genome, and miRBase database. Ribosomal RNA (rRNA), small nuclear RNA (snRNA), small nucleolar RNA (snoRNA), and transfer ribonucleic acid (tRNA); exon sense/exon antisense, intron sense/intron antisense might be fragments from mRNA degradation; repeat sequences include DNA transposon, long terminal repeat, long interspersed nuclear element, short interspersed nuclear element. osa-miRNA is rice miRNA included in miRBase. osa-miRNA edit means the base editing status of osa-miRNAs in our samples. Known microRNA (miRNA) refers to the miRNA of other species. Novel miRNA is miRNA predicted by software. Unannotation is unannotated clean reads.

The small RNA reads showed peaks at 21 and 24 nt (Figure 1A), a characteristic of plants with small RNAs size. All of the clean reads were aligned with the *O. sativa* reference genome (Nipponbare_Ensembl_IRGSP-1.0) and the *M. oryzae* reference genome (GCF_000002495.2_mg8), respectively. Out of all the clean reads, 75–90% were matched to the rice genome, 1–3% of the clean reads from the libraries of the inoculated samples matched the *M. oryzae* genome, while only about 0.05% of the clean reads in the libraries of the control samples matched the *M. oryzae* genome (Figure 1B). To identify the rRNA, scRNA, snoRNA, snRNA, and tRNA, all of the clean reads were aligned with the small RNAs in the GeneBank database (Release 209.0) and Rfam database (11.0), respectively. Those mapped to exons or introns might be fragments from mRNA degradation, and those mapped to repeat sequences (DNA transposons, long terminal repeat, long interspersed nuclear elements, short interspersed nuclear elements), were identified. Then, all of the clean reads were searched against the miRBase database (Release 21) to identify the rice miRNAs (osa-miRNA included in miRBase database), miRNA edit (Kawahara, 2012), and known miRNAs (the miRNAs can alignment with other species). In addition, we also predicted novel miRNAs from the unannotated data using the software Mireap_v0.2. Finally, we retrieved almost 30% of the miRNAs in total clean reads (Figure 1C, Supplementary Table 1). These results are similar to the previously reported small RNA-sequencing (RNA-seq) data of rice (Li et al., 2014; Zhang et al., 2018), indicating that further analysis can be made.

### Identification of miRNAs Differentially Expressed Upon *M. oryzae* Infection

**Figure 2:**
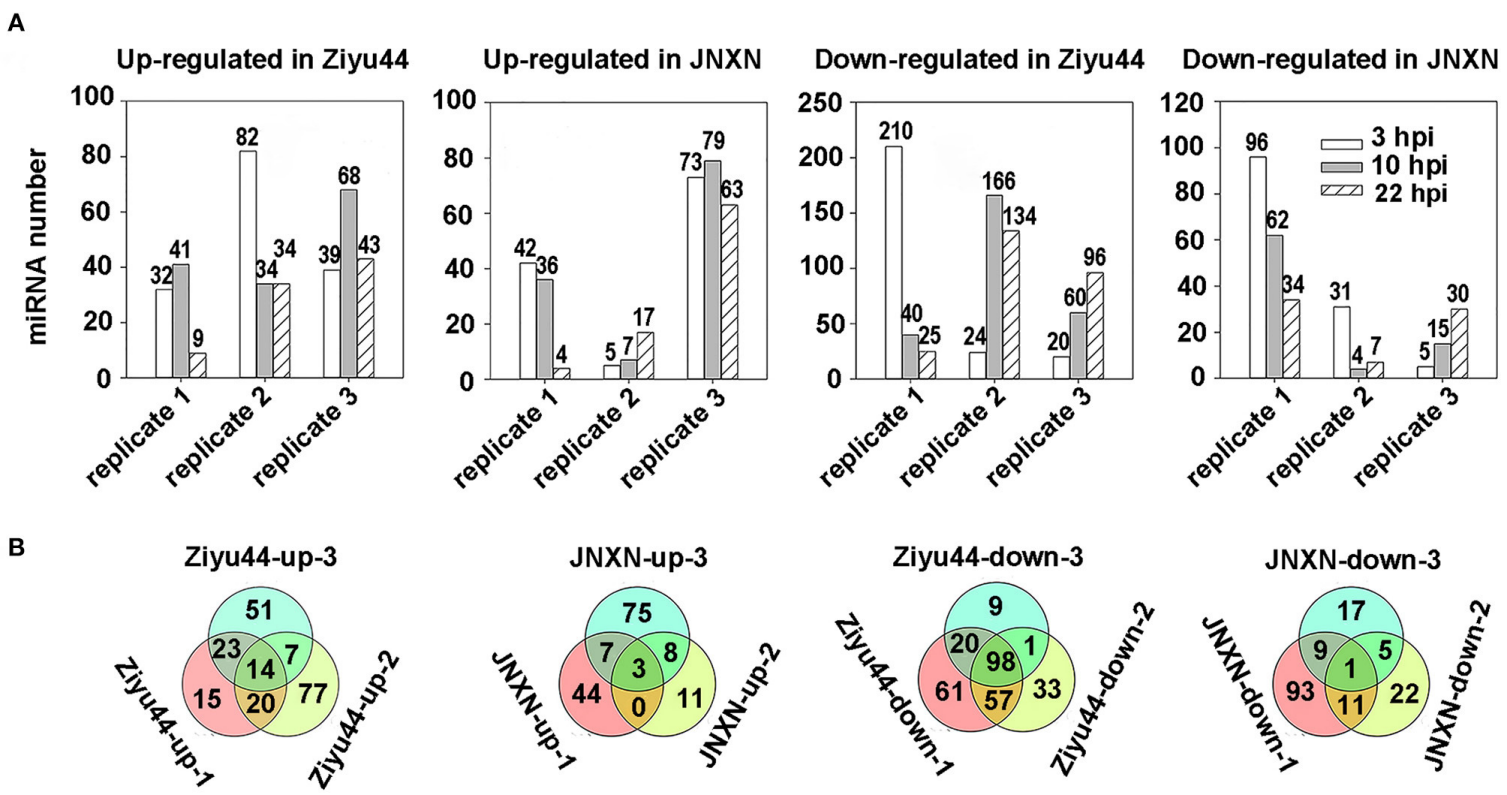
Analysis of the differentially expressed miRNAs with a two-fold variation. **(A)** Bar charts summarizing the number of differentially expressed miRNAs (treat/mock≥2 or treat/mock ≤0.5) in Ziyu44 and JNXN at every time point after being infected with *M. oryzae*. replicate 1, replicate 2, replicate 3 represents the three biological replicates performed three times under similar conditions. **(B)** The number of overlapped means differentially expressed miRNAs in Ziyu44 and JNXN among the three biological replicates. “Ziyu44-up-1” represents the number of all miRNAs upregulated at 3, 10, and 22 hpi in biological replicate 1, and so on.

To identify the miRNAs involved in the immunity of Ziyu44 against rice blast disease, we compared the expression levels of miRNAs in rice before and after the infection by *M. oryzae*. miRNAs both abundantly and significantly differentially expressed were screened by employing the following criteria: (1) Total reads ≥100, (2) treated/mock ≥2 (upregulated miRNAs), or treated/mock ≤ .5 (downregulated miRNAs) at three time points. In total, 466 miRNAs (upregulated 207, downregulated 259) and 306 miRNAs (upregulated 148, downregulated 158) with significantly different expressions were identified in Ziyu44 and JNXN, respectively (Figure 2, Supplementary Table 2). However, we found that the number and types of differentially expressed miRNAs have a large variation among biological replications (Figure 2A, Supplementary Table 2); the possible reasons will be discussed in the discussion section. In the following analysis, we focused on identifying the miRNAs that were constantly upregulated or downregulated among biological replications. In Ziyu44, 14 constantly upregulated and 98 constantly downregulated miRNAs were identified. In JNXN, three constantly upregulated and one constantly downregulated miRNAs were identified (Figure 2B). Upon *M. oryzae* infection, the number of constantly differentially expressed miRNAs in Ziyu44 was much higher than that in JNXN, especially the number of constantly downregulated miRNAs, indicating that more miRNAs respond to *M. oryzae* infection in Ziyu44 compared with the susceptible rice variety JNXN.

Some of the miRNAs with significantly different expressions are those that have been reported to regulate rice immunity against blast fungus (Table 1). The expression of osa-miR7695, osa-miR398b, and osa-miR166k/h were upregulated in the rice inoculated with *M. oryzae* isolates which positively regulated the rice immunity against rice blast disease (Campo et al., 2013; Salvador-Guirao et al., 2018; Li Y. et al., 2019). In our study, upon *M. oryzae* infection, osa-miR7695 was significantly upregulated in Ziyu44 and slightly downregulated in JNXN, while osa-miR398b was significantly up-regulated in Ziyu44 and slightly upregulated in JNXN (Table 1). Their expression patterns are consistent with previous studies (Campo et al., 2013; Li Y. et al., 2019), implying that osa-miR7695 and osa-miR398b may act as positive regulators to the resistance phenotype observed in Ziyu44. However, upon *M. oryzae* infection, the expression of osa-miR166k/h and the other members of the miR166 family were downregulated in Ziyu44 and slightly upregulated in JNXN, contrary to previous reports (Salvador-Guirao et al., 2018) (Table 1). The role of osa-miR166 might be affected by the genetic background of the rice materials. osa-miR156, osa-miR167d, osa-miR169, and osa-miR396 were reported as negative regulators in rice immunity against rice blast (Li Y. et al., 2017; Wang J. et al., 2018; Chandran et al., 2019; Zhao et al., 2019). In our study, upon *M. oryzae* infection, osa-miR156, osa-miR167d, osa-miR169, and osa-miR396 were significantly downregulated in Ziyu44 and upregulated in JNXN (Table 1), consistent with their negative roles as reported previously. In *Arabidopsis*, miR393 promoted basal defense against the virulent *Pseudomonas syringae* DC3000 (Pst DC3000) by silencing the F-box auxin receptors to suppress auxin signaling (Navarro et al., 2006). In our study, osa-miR393 was significantly upregulated in Ziyu44 upon *M. oryzae* infection (Table 1), denoting that it may be involved in the basal defense against *M. oryzae* and contribute to the broad-spectrum resistance in Ziyu44. Some differentially expressed miRNAs in Ziyu44 infected with rice blast fungus *M. oryzae* have not been reported to be involved in rice response to *M. oryzae* (Table 1); however, they might particularly contribute to immunity against rice blast disease in Ziyu44.

**Table 1:** Identified miRNAs with significant differential expression in Ziyu44.

	**Ziyu44**				**JNXN**			
		***M.oryzae*** **treated**				***M.oryzae*** **treated**		
**miRNA ID**	**0 h**	**3 h**	**10 h**	**22 h**	**0 h**	**3 h**	**10 h**	**22 h**
**Up-regulated in Ziyu44**								
osa-miR2877	21	108	71	67	50	19	35	32
osa-miR1432-3p	5	23	59	17	27	43	23	11
osa-miR7695-5p	63	43	206	90	69	61	57	38
osa-miR5082	39	97	137	258	53	55	49	28
osa-miR159f	501	962	772	1,377	997	473	485	345
osa-miR399j	24	166	236	224	125	79	90	64
osa-miR393b-3p	1,292	3,078	2,904	1,635	1,445	2,127	2,737	2,603
osa-miR408-3p	104	353	218	163	329	284	289	232
osa-miR528-5p	260	812	649	526	997	740	982	730
osa-miR5811	9	13	25	29	13	6	6	6
miR7767	45	74	106	95	81	48	35	35
osa-miR159a.1	164,035	283,867	280,782	397,187	296,298	160,730	153,545	149,179
osa-miR398b	35	113	88	159	80	98	139	119
**Down-regulated in Ziyu44**								
osa-miR156b-3p	554	254	215	79	35	145	165	99
osa-miR156h/f/l-3p	1,713	2,308	1,451	617	2,303	5,697	6,089	4,237
osa-miR156b-5p/c-5p/g-5p	8,707	7,389	4,353	5,933	2,677	10,221	10,240	8,191
osa-miR156c-3p	1,148	1,983	982	489	1,375	3,293	4,046	3,572
osa-miR156g-3p	1,149	1,985	984	491	1,377	3,294	4,051	3,577
osa-miR156l-5p	71	47	25	39	6	24	17	15
osa-miR1883a/b	230	89	55	74	80	112	120	138
osa-miR1862a/b/c	231	234	74	102	77	152	202	192
osa-miR1862d	2,961	4,121	2,222	1,382	1,136	1,561	1,665	1,531
osa-miR167h-3p	210	273	102	79	114	353	284	136
osa-miR167b	4,293	3,056	1,974	1,066	3,083	3,338	3,987	4,827
osa-miR167h-5p/d-5p/f/g/j	130,215	79,912	48,006	62,794	65,122	68,797	72,928	91,653
osa-miR167a-5p/c-5p	4,197	3,027	1,960	1,034	3,052	3,266	3,923	4,756
osa-miR167e-3p/i-3p	85	77	44	22	28	157	149	116
osa-miR167h-3p	210	273	102	79	114	353	284	136
osa-miR1878	160	159	75	40	49	126	116	141
osa-miR166i-3p	164	240	96	47	93	146	123	108
osa-miR166a/b/c/d-3p/f	16,508	27,178	11,945	7,610	14,551	15,884	18,738	18,537
osa-miR166g-3p	2,864	3,764	1,847	1,104	1,981	2,388	2,779	2,741
osa-miR166h-3p	1,428	1,971	795	467	963	1,243	1,450	1,477
osa-miR166k-3p/l-3p	5,561	4,633	2,005	1,577	2,970	4,458	5,233	5,388
osa-miR166m	814	845	434	213	554	728	890	682
osa-miR166a-5p	32	20	10	7	12	22	18	14
osa-miR166e-5p	30	18	9	7	11	21	17	13
osa-miR166j-5p	227	70	59	92	234	230	136	108
osa-miR396e-3p	95	40	26	30	35	171	166	141
osa-miR396f-3p	431	515	184	188	390	228	331	264
osa-miR396c-5p	13,621	12,409	6,470	3,031	7,848	14,254	14,960	12,668
osa-miR162a	1,716	2,397	1,551	850	1,003	1,633	1,717	1,659
osa-miR169e	637	231	249	291	102	109	158	77
osa-miR395	1,101	562	439	579	491	715	668	608
osa-miR1870-3p	82	54	46	26	63	89	81	85
osa-miR1846d-5p	32	23	15	16	14	27	38	32
osa-miR1849	98	105	77	39	77	118	96	95
osa-miR1856	46	30	19	24	41	93	94	122
osa-miR1863a	426	723	850	141	1,186	1,015	730	952
osa-miR1863b.2	46	70	34	21	21	26	29	24
osa-miR2864.1	48	59	36	22	31	89	81	61
osa-miR2871a-5p	78	76	33	31	29	54	41	47
osa-miR2874	21	24	28	9	32	31	26	33
osa-miR2878-3p	141	202	185	70	227	340	278	208
osa-miR2905	17	16	18	7	25	20	22	26
osa-miR396e-5p	168,798	155,797	85,359	65,905	127,244	142,906	150,462	174,831
osa-miR396f-5p	169,041	155,950	85,432	66,138	127,350	143,047	150,676	175,104
osa-miR5542	133	87	37	36	0	0	0	0
osa-miR5788	8,411	6,110	10,711	3,743	7,173	12,957	10,841	8,862
osa-miR7694-5p	36	43	18	17	22	41	42	44
osa-miR810b.1	391	278	255	128	282	642	481	380
osa-miR812r	20	14	14	9	17	20	23	25
osa-miR827	188	230	248	62	321	325	267	259
**No miRNA level in Ziyu44**								
osa-miR1882a/b/c/d/e-5p/f/g/h	0	0	0	1	142	260	313	278
osa-miR812i/h/j/g/p	2	1	1	4	159	67	103	138
osa-miR439a/b/c/e/f/g/h	1	1	1	0	4	27	19	10
**Only expressed in Ziyu44**								
osa-miR3980b-5p	131	381	662	343	0	0	0	0
osa-miR3980b-3p	40	17	27	28	0	0	0	0
osa-miR3980a-5p	131	381	662	343	0	0	0	0
osa-miR3980a-3p	40	17	27	28	0	0	0	0
**Up-regulated in Ziyu44 and JNXN**								
osa-miR1320-5p	21	116	94	65	26	107	125	96
osa-miR1320-3p	100	205	232	168	79	245	421	585
osa-miR169i-5p.2	892	772	2,568	2,516	649	737	1,765	1,624
miR9664	37	74	145	108	52	94	79	60
**Down-regulated in Ziyu44 and JNXN**								
osa-miR166j-5p	272	172	47	40	212	83	75	91

*miRNAs with more than 2-fold variation are listed. Reads are normalized to library size and are presented in read per million (RPM)*.

### Reverse-Transcription Quantitative PCR Verified the Small RNA-Seq Data

**Figure 3:**
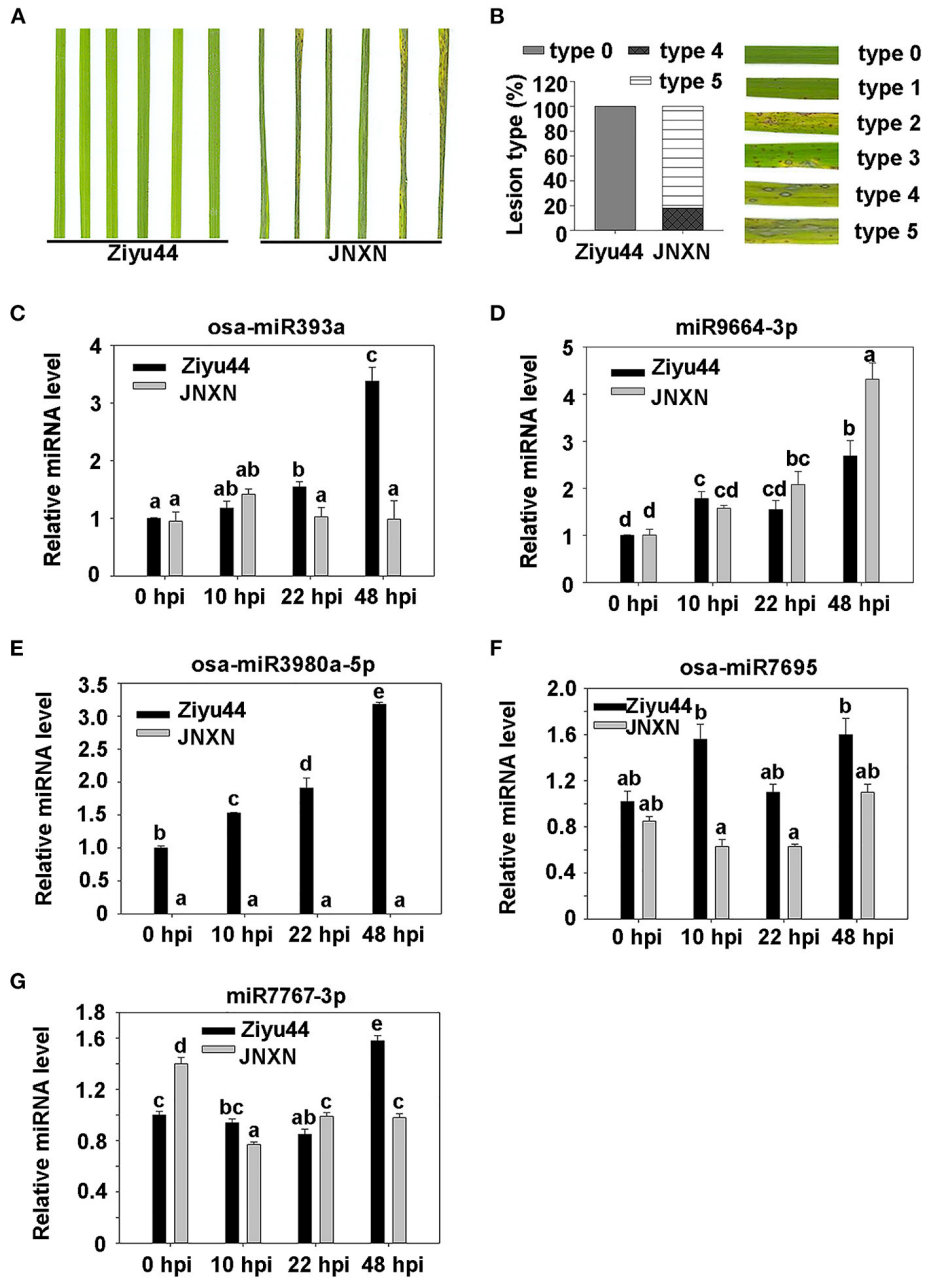
Validation of some significantly differentially expressed miRNAs by RT-qPCR. **(A)** The phenotype of the leaf sections from the resistant accession Ziyu44 and susceptible accession JNXN inoculated with *M. oryzae* at 5 dpi. **(B)** The representative phenotype of lesion types, and the percentage of the Ziyu44 and JNXN lesion types. **(C–G)** Expression pattern of osa-miR393a **(C)**, miR9664 **(D)**, osa-miR3980a-5p **(E)**, osa-miR7695 **(F)**, and miR7767-3p **(G)** in Ziyu44 and JNXN after being infected with *M. oryzae*. miRNA level is normalized to that in the untreated control plants (0 hpi). Error bars indicate SD (*n* = 3). The letters above the bars indicate significant differences at a value of *P* < 0.01 as determined by a one-way ANOVA followed by a *post-hoc* Tukey HSD analysis. Similar results were obtained from the three independent experiments.

To validate the small RNA-Seq data, four-leaf-stage seedlings of Ziyu44 and JNXN were inoculated with a conidial mixture of four *M. oryzae* strains (81278, ZB13, TC61, and H63) selected randomly from the 10 strains used in performing small RNA-seq. The disease phenotype was recorded at 5 dpi and Ziyu44 displays high resistance to the *M. oryzae* strains mixture (Figures 3A,B). Infected leaves were collected at 10, 22, and 48 hpi, with the leaves before inoculation as a control (0 hpi). Five miRNAs were reverse transcribed using the stem-loop method (Chen et al., 2005), respectively, and were used for qRT-PCR analysis. Upon being infected by *M. oryzae*, osa-miR393a was slightly upregulated at 10 and 22 hpi and was significantly upregulated at 48 hpi in Ziyu44 (Figure 3C). miR9664 was induced upon blast infection in both Ziyu44 and JNXN, but the relative expression level was upregulated slightly in Ziyu44 and drastically in JNXN, especially at 24–48 hpi (Figure 3D). osa-miR3980a-5p was specifically upregulated in Ziyu44 but did not accumulate in JNXN (Figure 3E), while osa-miR7695 was significantly upregulated at 10 and 48 hpi in Ziyu44, and was downregulated in JNXN (Figure 3F). miR7767-3p was significantly up-regulated at 48 hpi in Ziyu44 and downregulated in JNXN (Figure 3G). Their expression patterns are consistent with the small RNA-seq data. It is also similar to the reported results that the expression of miR393 was induced by the PAMP molecule flg22 in *Arabidopsis* (Navarro et al., 2006), and that miR7695 overexpressed rice plants conferred resistance to *M. oryzae* (Campo et al., 2013). These results indicate that our small RNA-seq data is reliable.

### Prediction and Analysis of miRNA Target Genes

**Figure 4:**
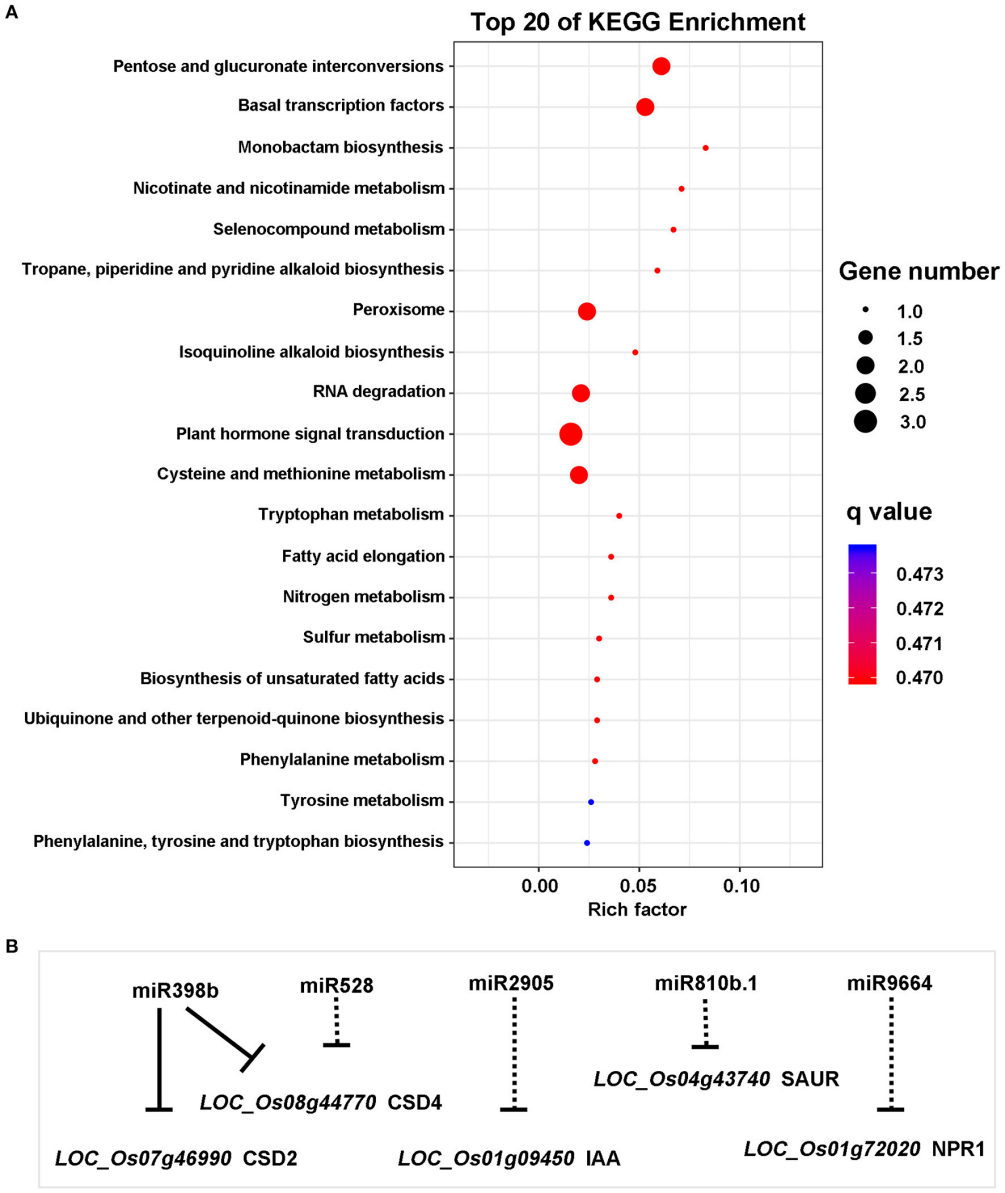
Analysis of predicted target genes. **(A)** The top 20 Kyoto Encyclopedia of Genes and Genomes (KEGG) pathway terms. The ordinates in the figure are the KEGG's B-level categories, which is the specific pathway name. The abscissa is the rich factor, which refers to the degree of enrichment. The size of the circle represents the number of genes. The color represents the *Q*-value (corrected-*P*-value). **(B)** miRNAs and the corresponding target genes (solid line)/predicted target genes (dotted line) in the main disease resistance pathway.

To further explore the role of miRNAs in the broad-spectrum resistance to rice blast in Ziyu44, we predicted target genes with significantly differentially expressed miRNAs using the online software psRNATarget (http://plantgrn.noble.org/psRNATarget/), in which we selected two relatively authoritative databases, Michigan State University - Rice Genome Annotation Project Database (MSU) and Rice Annotation Project Database (RAP) as the target transcript library respectively. By converting the gene IDs from RAP and MSU, we found that the predicted target genes searched from the two databases are similar (Supplementary Table 3). In total, we obtained 467 candidate target genes from the two databases (Supplementary Table 3). Except for two miRNAs, including osa-miR1846d-5p and osa-miR166j-5p, which have no predicted target genes, the other miRNAs have one or more predicted target genes (Supplementary Table 3). To learn about the possible biological functions of these candidate genes, a pathway enrichment analysis was performed using OmicShare tools (www.omicshare.com/tools), a free online platform for data analysis. Forty-four predicted target genes were enriched in the top 20 Kyoto Encyclopedia of Genes and Genomes (KEGG) pathway terms (Supplementary Figure 1). Among them, three genes*, namely, LOC_Os01g09450, LOC_Os04g43740*, and *LOC_Os01g72020* were enriched in the plant hormone signal transduction pathway, and two genes, namely, *LOC_Os07g46990* and *LOC_Os08g44770* were enriched in the peroxisome pathway (Figure 4), which is related to plant disease resistance. Next, we analyzed the expression patterns of the five genes above through the corresponding mRNA sequencing data (our unpublished data) and found that the expression of *LOC_Os04g43740* was not detected in both Ziyu44 and JNXN and the other genes displayed a similar expression pattern between Ziyu44 and JNXN upon *M. oryzae* infection (Table 2). The RT-PCR results were similar to the transcriptome data (Supplementary Figure 2).

**Table 2:** Expression pattern of 5 predicted target genes enriched in plant hormone signal transduction and peroxisome pathway.

**target gene ID^a^**	**miRNA ID^b^**	**The expression level of predicted target genes^c^**								**Description^d^**
		**Ziyu44**				**JNXN**				
		**0 h**	**3 h**	**10 h**	**22 h**	**0 h**	**3 h**	**10 h**	**22 h**	
*LOC_Os01g09450*	osa-miR2905	5	5	3	3	8	7	4	2	IAA
*LOC_Os04g43740*	osa-miR810b.1	0	0	0	0	0	0	0	0	SAUR
*LOC_Os01g72020*	miR9664	12	12	22	18	12	10	18	16	NPR1
*LOC_Os07g46990*	osa-miR398b	150	175	301	337	167	154	231	366	CSD
*LOC_Os08g44770*	osa-miR528	20	25	20	22	17	13	12	12	CSD

a *LOC_Os01g09450, LOC_Os04g43740, and LOC_Os01g72020 were enriched in the plant hormone signal transduction pathway, LOC_Os07g46990 and LOC_Os08g44770 were enriched in the peroxisome pathway*.

b *The corresponding miRNAs of predicted target genes are listed in this table*.

c *Reads are normalized to library size and are presented in reads per million (RPM)*.

d *Description of the gene product. LOC_Os01g09450: OsIAA2–Auxin-responsive Aux/IAA gene family member, expressed; LOC_Os04g43740: OsSAUR18–Auxin-responsive SAUR gene family member, expressed; LOC_Os01g72020: BTBA3–Bric-a-Brac, Tramtrack, Broad Complex BTB domain with Ankyrin repeat region, regulatory protein NPR1, expressed; LOC_Os07g46990: copper/zinc superoxide dismutase, putative, expressed; LOC_Os08g44770: copper/zinc superoxide dismutase, putative, expressed*.

As described above, we could only identify very few genes that may contribute to the resistance phenotype in Ziyu44 by analyzing the predicted target genes enriched in the KEGG pathway, so we further used the corresponding mRNA-seq data to analyze the expression patterns of all the predicted target genes (Supplementary Table 3). In total, 118 predicted target genes with two-fold variation (treat/mock ≥ 2 or treat/mock ≤ 0.5) were identified. Among them, 24 genes were specifically upregulated in Ziyu44, 15 genes were specifically downregulated in Ziyu44, 5 genes were specifically upregulated in JNXN, 9 genes were specifically downregulated in JNXN, 30 genes were upregulated in both Ziyu44 and JNXN, 33 genes were downregulated in both Ziyu44 and JNXN, 1 gene was upregulated in Ziyu44 and down-regulated in JNXN, and 1 gene was downregulated in Ziyu44 and upregulated in JNXN (Supplementary Figure 3). Among them, 56 genes with relatively high expression levels (total reads ≥10) were listed in Supplementary Table 4. Although the expression patterns of most of the predicted target genes were not negatively correlated with their corresponding miRNAs as expected, some genes with significantly different expressions between Ziyu44 and JNXN are disease resistance-related (Table 3). Further functional studies of these genes might help us understand the mechanism of rice blast resistance in Ziyu44.

**Table 3:** Significantly differentially expressed predicted target genes.

**Target gene ID**	**miRNA ID**	**Gene product name**
LOC_Os02g39070	osa-miR1862d	Vesicle transport protein GOT1B, putative, expressed
LOC_Os08g01390	osa-miR3980a-5p	Phosphatidylinositol-4-phosphate 5-Kinase, putative, expressed
LOC_Oso8g30780.1	osa-miR2905	ABC transporter, ATP-binding protein, putative, expressed
LOC_Os05g40170.1		Expressed protein
LOC_Os01g14100.1		BT1 family protein, putative, expressed
LOC_Os02g50470.1		Expressed protein
LOC_Os08g33710.1		Ribonuclease T2 family domain containing protein, expressed
LOC_Os01g09450.1		OsIAA2–Auxin-responsive Aux/IAA gene family member, expressed
LOC_Oso8g31780.1	miR9664-3p	MLA1, NB-ARC domain containing protein, putative, expressed
LOC_Os02g18510.1		Similar to Stripe rust resistance protein Yr10, NB-ARC domain containing protein
LOC_Os11g45924.1		WRKY41, NB-ARC domain containing protein, expressed
LOC_Os02g17304.1		Resistance protein, NB-ARC domain containing protein, putative, expressed
LOC_Os11g34920.1		Stripe rust resistance protein Yr10, NB-ARC domain containing protein, putative, expressed
LOC_Os08g42700.1		Resistance protein, NB-ARC domain containing protein, putative, expressed
LOC_Os08g29809.1		Resistance protein LR10, NB-ARC domain containing protein, putative, expressed
LOC_Os02g08364.1		Protein phosphatase 2C, NB-ARC domain containing protein, putative, expressed
LOC_Os10g02380.1		Probable NAD(P)H-dependent oxidoreductase
LOC_Os12g36720.1		RGH1A, NB-ARC domain containing protein, putative, expressed
LOC_Os12g37760.1		RGH1A, NB-ARC domain containing protein, putative, expressed
LOC_Os06g17970.1		NBS-LRR disease resistance protein, putative, expressed
LOC_Os08g07774.1		Disease resistance protein RPM1, NB-ARC domain containing protein, putative, expressed
LOC_Os12g17090.1		Stripe rust resistance protein Yr10, NB-ARC domain containing protein, putative, expressed
LOC_Os01g68770.1	osa-miR398b	Selenium-binding protein, putative, expressed
LOC_Os07g46990.1		Copper/zinc superoxide dismutase, putative, expressed
LOC_Os01g66760	osa-miR5542	Inactive receptor kinase At2g26730 precursor, putative, expressed
LOC_Os03g45410.1		TATA-binding protein, putative, expressed
LOC_Os04g45580.1	osa-miR167h-3p	Kinesin motor domain containing protein, expressed
LOC_Os08g40230.1	osa-miR396c-5p	Expressed protein
LOC_Oso1g59660	osa-miR159f/a.1	MYB family transcription factor, putative, expressed
LOC_Os03g21380.1		OsCML27–Calmodulin-related calcium sensor protein, expressed
LOC_Os09g39740.1		ACT domain containing protein, expressed
LOC_Os07g07260.1	osa-miR2874	Glutamine-dependent NAD, putative, expressed
LOC_Os02g53180.1	osa-miR812g	1-aminocyclopropane-1-carboxylate oxidase protein, putative, expressed
LOC_Os08g04310.1	osa-miR528-5p	Plastocyanin-like domain containing protein, putative, expressed
LOC_Os01g17150.1		Plastid-Specific 50S ribosomal protein 5, chloroplast precursor, putative, expressed
LOC_Oso4g48390.1	osa-miR827	Uncharacterized membrane protein, putative, expressed
LOC_Os02g56370.1		OsWAK20–OsWAK receptor-like protein kinase, expressed
LOC_Os02g45520.1		Uncharacterized membrane protein, putative, expressed
LOC_Os05g34460.1	osa-miR166i-3p	OsDegp7–Putative Deg protease homolog, expressed
LOC_Os03g43930.1		START domain containing protein, expressed
LOC_Os04g46570.1		Growth regulator related protein, putative, expressed
LOC_Os06g03830.1	osa-miR167a-5p	Retinol dehydrogenase, putative, expressed
LOC_Oso1g51800	osa-miR5788	Expressed protein
LOC_Os09g22505.1		Expressed protein
LOC_Os11g02400.1		LTPL8–Protease inhibitor/seed storage/LTP family protein precursor, expressed
LOC_Os09g31478.1	osa-miR7695-5p	Auxin efflux carrier component, putative, expressed
LOC_Os03g02550.1	osa-miR393b-3p	OsFBX76–F-box domain containing protein, expressed
LOC_Os05g45350.1	osa-miR399j	dnaJ domain containing protein, expressed
LOC_Os04g55230.1		Tetratricopeptide repeat domain containing protein, putative, expressed
LOC_Oso6g49010.1	osa-miR156b	OsSPL12–SBP-box gene family member, expressed
LOC_Os02g04680.1		OsSPL3–SBP-box gene family member, expressed
LOC_Os05g38590.1	osa-miR1320-3p	Expressed protein
LOC_Os07g41720.1	osa-miR169e	Nuclear transcription factor Y subunit, putative, expressed
LOC_Os03g29760.1		Nuclear transcription factor Y subunit, putative, expressed
LOC_Os03g48970.1		Nuclear transcription factor Y subunit, putative, expressed
LOC_Os09g10274.1	osa-miR1856	Expressed protein

### miR9664 Negatively Regulates Blast Disease Resistance

**Figure 5:**
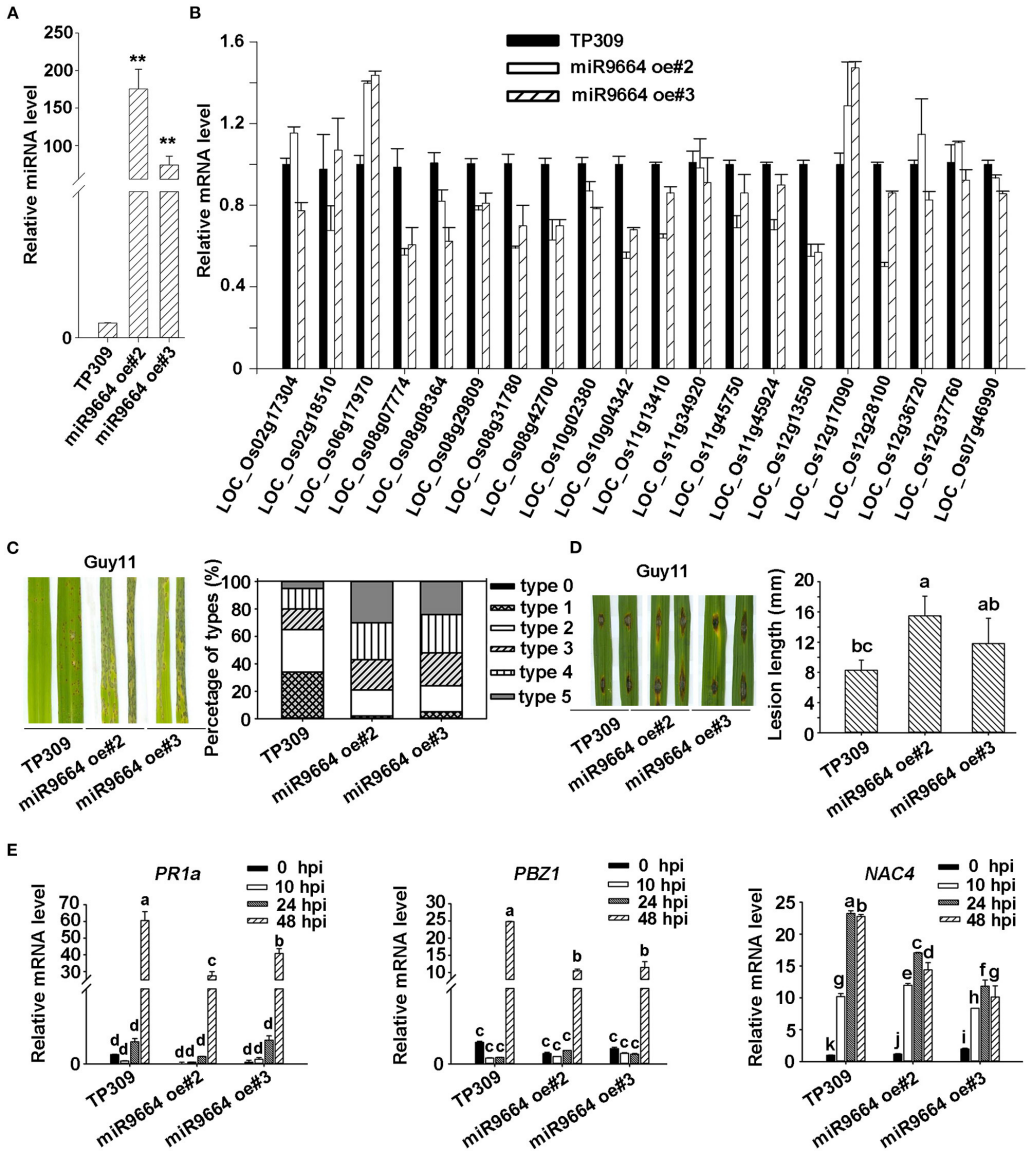
miR9664-oe plants enhanced susceptibility to *M. oryzae* GUY11. **(A)** The relative expression level of miR9664 in the two miR9664-oe lines. The ** above the bars indicate significant differences (*P* < 0.01). **(B)** The RNA expression level of 19 miR9664 predicted target genes in the two miR9664-oe lines, the non-target gene *LOC_Os07g46990* was used as a control gene, qRT-PCR results were normalized with the Ubi reference gene. Similar results were obtained from the three independent biological experiments. **(C)** The blast resistance of miR9664-oe plants using spraying inoculation. Two inoculated leaves of each TP309, miR9664 oe#2, and miR9664 oe#3 are shown. The lesion types were obtained from the statistics of 80 diseased leaves at 5 dpi. **(D)** The blast resistance of miR9664-oe plants using punching inoculation. Two inoculated leaves of each TP309, miR9664 oe#2, and miR9664 oe#3 are shown. The lesion length was measured using a caliper. Error bars indicate SD (*n* = 12), the letters above the bars indicate significant differences (*P* < 0.01). **(E)** RNA expression levels of the defense-related gene *PR1a, PBZ1*, and *NAC4* in miR9664-oe plants, qRT-PCR results were normalized with the Ubi reference gene, relative mRNA levels were normalized to those in the untreated control plants (0 hpi). Error bars represent the SD of three replicates (*n* = 3). The letters above the bars indicate significant differences at a value of *P* < 0.01 as determined by a one-way ANOVA followed by a *post-hoc* Tukey HSD analysis. Similar results were obtained from the three independent biological experiments.

**Figure 6:**
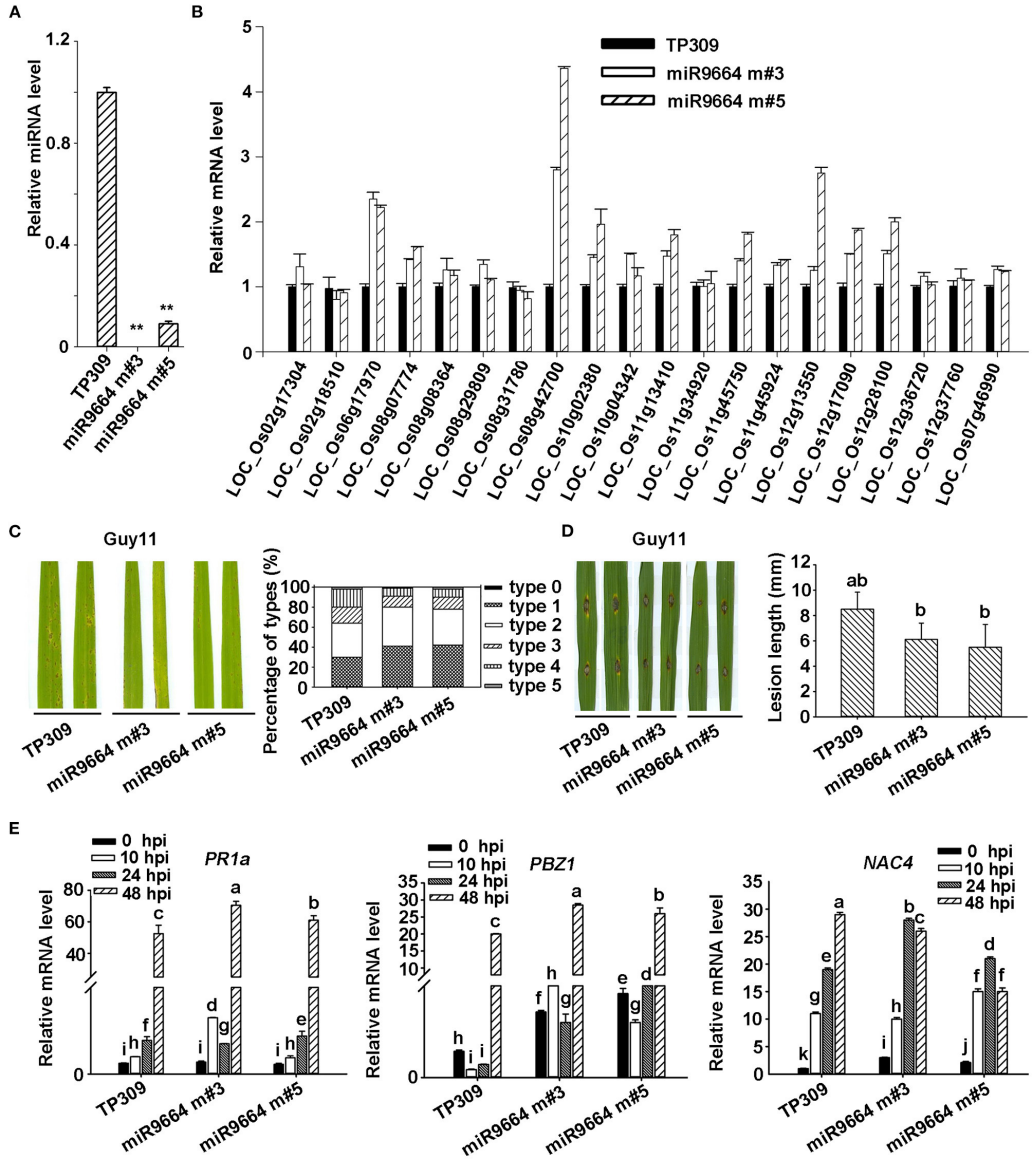
miR9664-m plants enhance resistance to *M. oryzae* GUY11. **(A)** The relative expression level of miR9664 in two miR9664-m lines. The ** above the bars indicate significant differences (*P* < 0.01). **(B)** The RNA expression level of 19 miR9664 predicted target genes in the two miR9664-oe lines, the non-target gene *LOC_Os07g46990* was used as a control, qRT-PCR results were normalized with the Ubi reference gene. Similar results were obtained from the three independent biological experiments. **(C)** The blast resistance of miR9664-m plants using spraying inoculation. Two inoculated leaves of each TP309, miR9664 m#3, and miR9664 m#5 are shown. The lesion types were obtained from the statistics of 80 diseased leaves at 5 dpi. **(D)** The blast resistance of miR9664-m plants using punching inoculation. Two inoculated leaves of each TP309, miR9664 m#3, and miR9664 m#5 are shown. The lesion length was measured using a caliper. Error bars indicate SD (*n* = 12), the letters above the bars indicate significant differences (*P* < 0.01). **(E)** RNA expression levels of the defense-related gene *PR1a, PBZ1*, and *NAC4* in miR9664-m plants, qRT-PCR results were normalized with the Ubi reference gene, the indicated mRNA levels were normalized to those in the untreated control plants (0 hpi). Error bars represent the SD of three replicates (*n* = 3). The letters above the bars indicate significant differences at a value of *P* < 0.01 as determined by a one-way ANOVA followed by a *post-hoc* Tukey HSD analysis. Similar results were obtained from the three independent biological experiments.

Increasing evidence showed that plant *R* genes are under direct targeting by miRNAs (Ouyang et al., 2014; Baldrich and San Segundo, 2016; Deng et al., 2018; Prigigallo et al., 2019; Zhang R. et al., 2019; Zhang Y. et al., 2019; Cui et al., 2020). Although many other different expression miRNAs identified in Ziyu44 may play a bigger role in the defense against the blast disease, such as osa-miR7695 (Campo et al., 2013), osa-miR398b (Li Y. et al., 2019), osa-miR167 (Zhao et al., 2019) and osa-miR169 (Li Y. et al., 2017), miR9664 is 22 nt, not documented in the previous rice miRNA database, could alignment with tae-miR9664 (*Triticum aestivum* L), and what's more, except for *LOC_Os10g02380.1* encoding a probable NAD(P)H-dependent oxidoreductase, all the other predicted target genes of miR9664 encode proteins containing NB-ARC domain (Table 3, Supplementary Table 3), so we focused on the functional studies of miR9664 in this study. To investigate the function of miR9664, we generated transgenic plants by the knockdown (miR9664-m) or overexpression (miR9664-oe) of miR9664 under the TP309 background (Figures 5A, 6A). We observed that the miR9664-oe plants developed larger lesions and displayed more severe disease symptoms compared to the wild type (TP309) (Figures 5C,D, Supplementary Figure 4), while the miR9664-m plants developed smaller lesions and showed reduced disease symptoms compared to TP309 (Figures 6C,D, Supplementary Figure 4). Further, we carried out an expression analysis of 19 predicted target genes of miR9664; the non-target gene *LOC_Os07g46990* was used as a control gene. We found that the expression levels of *LOC_Os08g31780, LOC_Os08g42700, LOC_Os08g07774, LOC_Os10g04342*, and *LOC_Os12g13550* were decreased in both two miR9664-oe lines compared with the control gene *LOC_Os07g46990* (Figure 5B, Supplementary Figure 5), while the expression levels of *LOC_Os06g17090, LOC_Os08g42700, LOC_Os08g07774, LOC_Os10g02380, LOC_Os11g13410, LOC_Os11g45750, LOC_Os12g13550, LOC_Os12g17970*, and *LOC_Os12g28100* were increased in both miR9664-m lines compared with the control gene *LOC_Os07g46990* (Figure 6B, Supplementary Figure 6). At the same time, we noticed that the expression of *LOC_Os08g42700, LOC_Os08g07774*, and *LOC_Os12g13550* was significantly down-regulated in two miR9664-oe lines while it was significantly induced in two miR9664-m lines, which suggested that they may be the major targets of miR9664. Besides, the expression levels of the defense-related marker genes, including *PR1a* (*pathogenesis-related 1a*), *PBZ1*(*PR10a*), and *NAC4* (Li et al., 2014), were significantly decreased in the miR9664-oe lines (Figure 5E) and increased in the miR9664-m lines (Figure 6E) compared with TP309. These results indicate that miR9664 is a novel rice miRNA negatively regulating rice immunity against the blast *M. oryzae* fungus.

**Figure 7:**
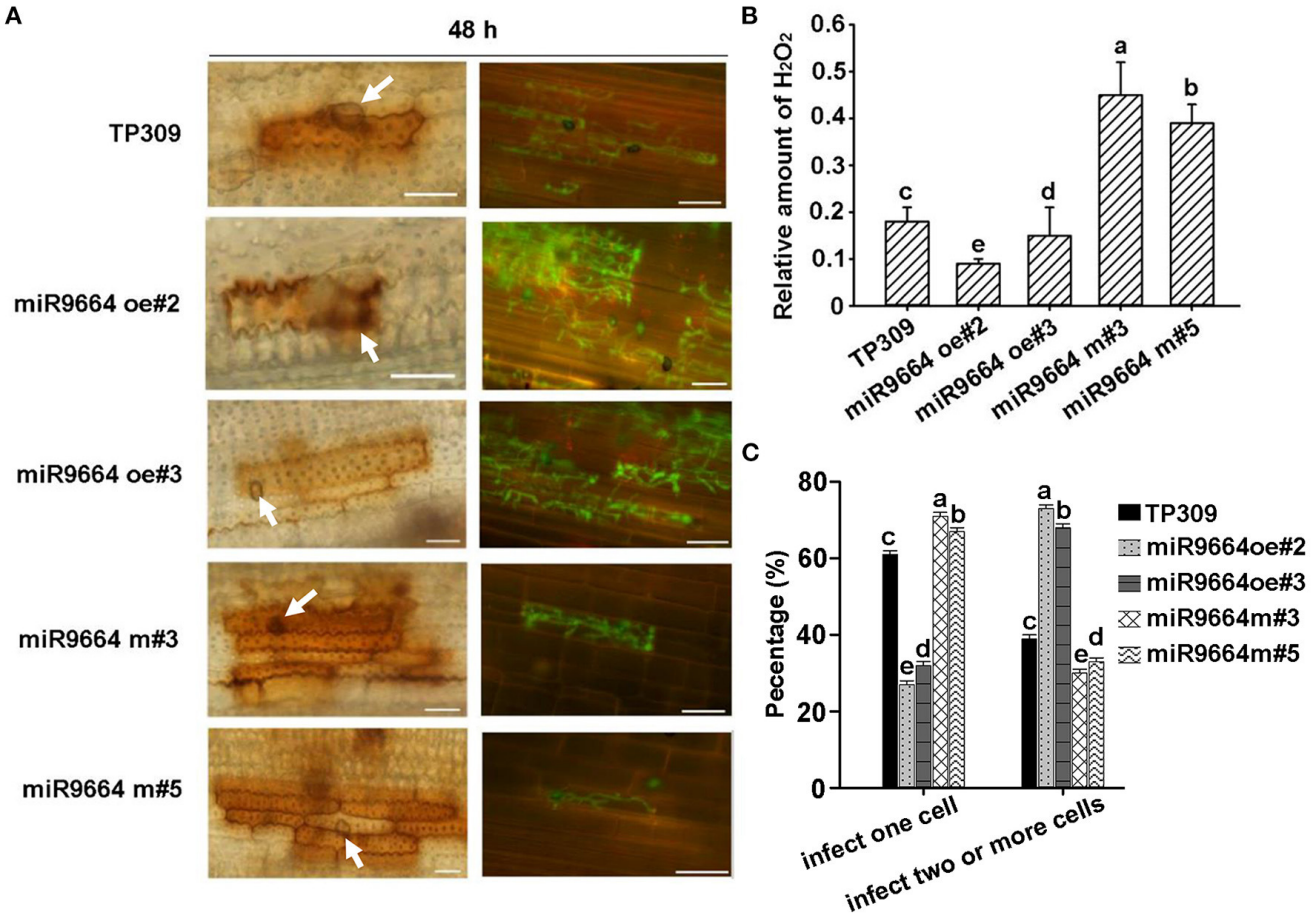
Cellular responses to *M. oryzae* infection. **(A)** Diaminobenzidine (DAB) staining at the infection sites of TP309, miR9664-oe, and miR9664-m plants at 2 dpi (left). The tawny shading indicates the accumulation of hydrogen peroxide (H_2_O_2_), the arrows indicate the infection structure of appressoria; Representative laser scanning microscopy images of TP309, miR9664-oe, and miR9664-m sheath cells infected by the eGFP-tagged blast isolate Zhong 10-8-142 dpi (right). Scale bars, 20 μm. **(B)** Quantitation of H_2_O_2_ shown in **(A)** left. The relative amount of H_2_O_2_ was calculated based on the pixels taken with Photoshop, using the formula: H_2_O_2_ area per rectangle = pixel of H_2_O_2_ area per leaf/pixel of the rectangle. Error bars represent the SD of three replicates (*n* = 3), the letters above the bars indicate significant differences (*P* < 0.01). **(C)** Quantitative analysis of *M. oryzae* growth at 48 hpi. More than 60 conidia in each line were analyzed. Error bars represent SD (*n* = 60), the letters above the bars indicate significant differences (*P* < 0.01). All of the experiments were repeated three times with similar results.

We also examined the plant response at a cellular level to investigate the cellular mechanism of miR9664 negatively regulating rice blast disease resistance. Staining the inoculated leaf cells with 3,3′-diaminobenzidine (DAB) for hydrogen peroxide (H_2_O_2_) reveals that miR9664-oe plants produce a lower amount of H_2_O_2_ in the inoculated leaf cells than TP309, while miR9664-m plants produce a higher amount of H_2_O_2_ in the inoculated leaf cells than TP309 (Figures 7A,B). Consistent with the DAB staining results, we observed that the invaded hyphae of GFP-tagged strain zhong-10-8-14(GZ8) substantially extended to multiple sheath cells of miR9664-oe plants at 48 hpi, while the hyphae extension was greatly restricted in the sheath cells of miR9664-m plants and TP309 (Figures 7A,C). The result indicates that reduced ROS burst is a part of the mechanism of miR9664 in negatively regulating rice immunity to *M. oryzae*.

### miR9664-Mediated *R* Genes Turnover Contribute to Ziyu44 Broad-Spectrum Resistance to Rice Blast Fungus

**Figure 8:**
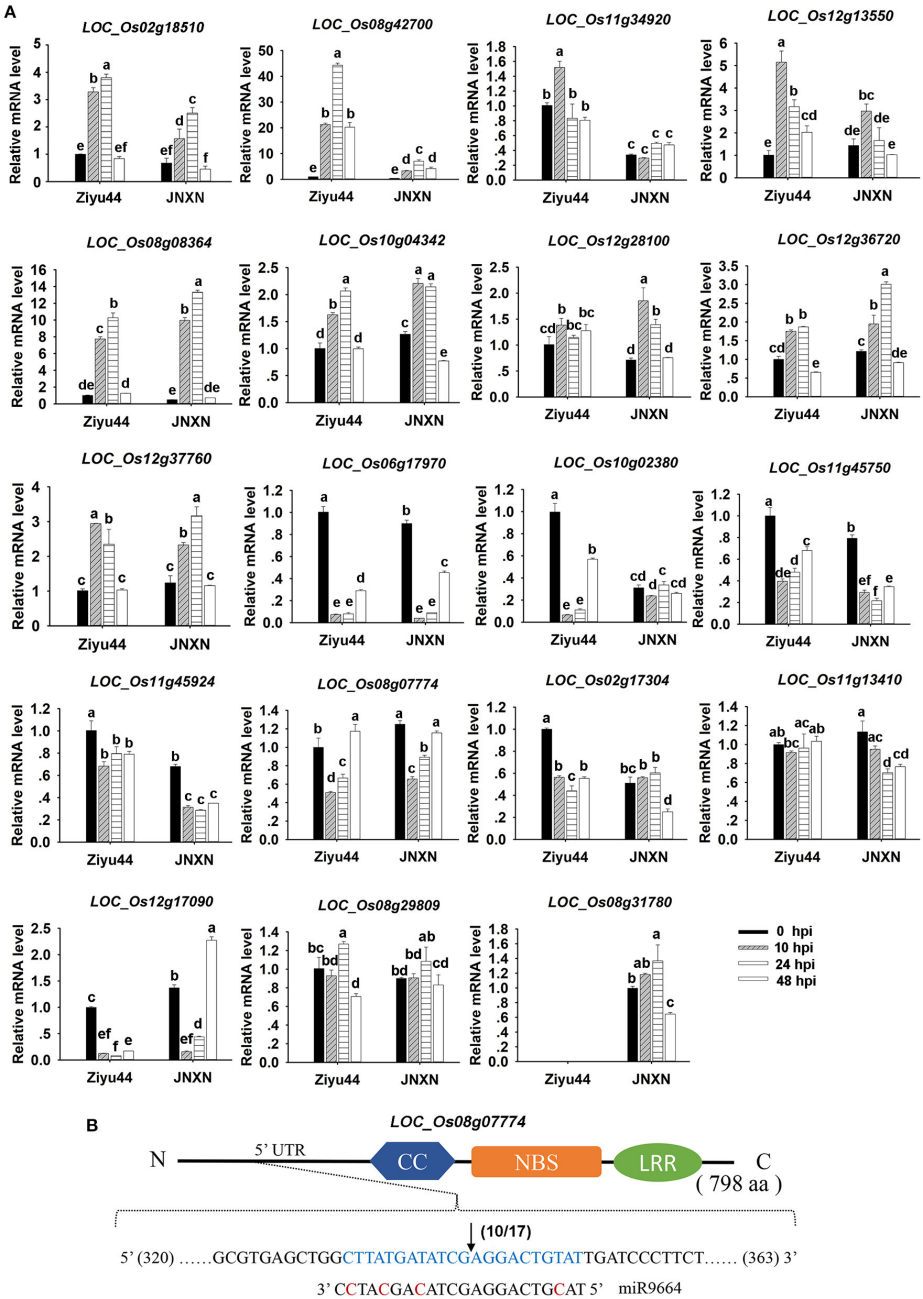
**(A)** Expression of miR9664 target genes in Ziyu44 and JNXN upon *M. oryzae* infection. qRT-PCR results were normalized with the Ubi reference gene, the indicated mRNA level is normalized to those in the untreated control plants (0 hpi). Error bars indicate SD (*n* = 3), the letters above the bars indicate significant differences (*P* < 0.01). Similar results were obtained from the three independent experiments. **(B)** 5′-RACE assays showing the cleavage sites within miR9664 target regions of the target genes *LOC_Os08g07774*. The cleavage site is shown with a black arrow, 10 out of 17 monoclonal sequencings showed the same results. The predicted CC, NBS, and LRR domains encoded by the target genes are labeled. N and C represent the amino and carboxyl terminals of R proteins, respectively. aa, amino acid.

To obtain more information on 19 predicted target genes of miR9664, we performed a time course examination of their expressions in Ziyu44 and JNXN upon *M. oryzae* infection (Figure 8A). The results showed that the expression patterns of these genes were varied between Ziyu44 and JNXN. The expressions of *LOC_Os02g18510, LOC_Os08g42700, LOC_Os11g34920, LOC_Os12g13550, LOC_Os08g08364, LOC_Os10g04342, LOC_Os12g28100, LOC_Os12g36720, LOC_Os12g37760*, and *LOC_Os08g29809* were induced by blast infection in both Ziyu44 and JNXN, especially at 10–24 hpi, but the induced expression levels of *LOC_Os02g18510, LOC_Os08g42700, LOC_Os11g34920*, and *LOC_Os12g13550* were significantly higher in Ziyu 44 than in JNXN. Although the expression level of *LOC_Os08g07774* was upregulated in Ziyu44 at 48 hpi, the expression level and pattern were very similar in the two rice cultivars. Except for *LOC_Os08g31780* whose expression was not detected in Ziyu 44, the expression levels of the other eight genes were downregulated in both Ziyu44 and JNXN, especially at 10–24 hpi. Additionally, the expression of miR9664 was induced by blast infection in both Ziyu44 and JNXN; slightly in Ziyu44 and drastically in JNXN, especially at 24–48 hpi (Figure 3).

Furthermore, we carried out an analysis on the miR9664-mediated cleavage of 13 predicted target genes (*LOC_Os02g17304, LOC_Os08g07774, LOC_Os08g29809, LOC_Os08g31780, LOC_Os08g42700, LOC_Os10g04342, LOC_Os11g13410, LOC_Os11g34920, LOC_Os11g45750, LOC_Os11g45790, LOC_Os12g17090, LOC_Os12g28100*, and *LOC_Os12g37760*) in the miR9664-oe#2 line using a 5′RLM-RACE assay. However, a specific cleavage within the miR9664 target site was detected in only the predicted gene *LOC_Os08g07774* (Figure 8B, Supplementary Figure 7). The other genes, like *LOC_Os08g31780, LOC_Os10g04342, LOC_Os12g17090*, and *LOC_Os08g42700* were truncated as expected, but the cleavage sites were not matched to the miR9664 complementary sequences (Supplementary Figure 7A).

In summary, we suggest that miR9664 could influence the expression levels of its predicted target genes. The slight upregulation of miR9664 is conducive to disease resistance in Ziyu44, a sharp upregulation will lead to rice susceptibility, and miR9664 guides the sequence-specific cleavage of *LOC_Os08g07774* in rice. The targeting relationship of other genes with miR9664 needs to be further confirmed.

## Discussion

**Figure 9:**
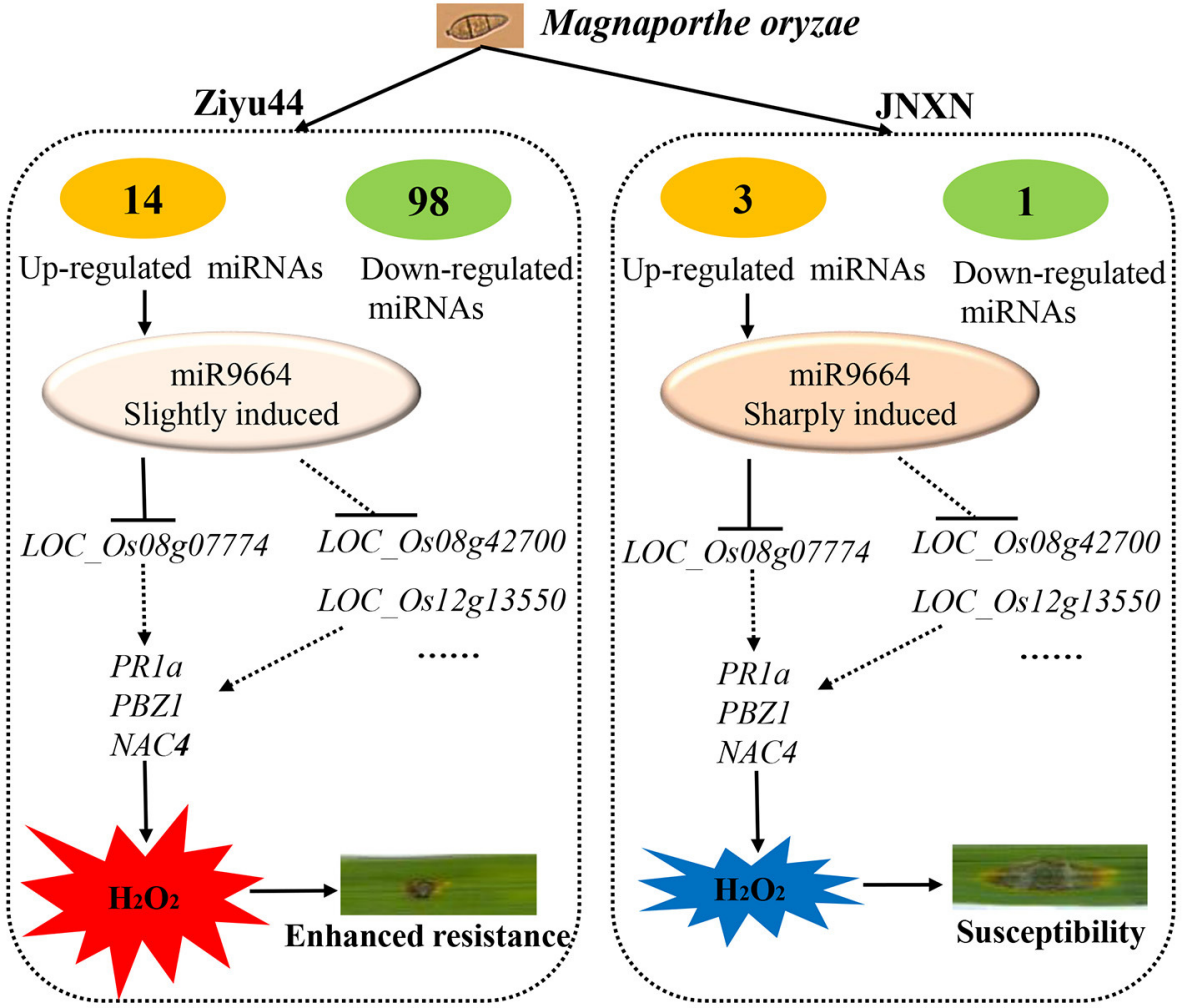
A model for miRNAs mediated rice blast resistance in rice. The number of differentially expressed miRNAs in the resistant cultivar Ziyu44 is more than that in the susceptible cultivar JNXN infected with *M. oryzae*. miR9664, a novel rice miRNA identified in this study, is induced in both Ziyu44 and JNXN, however, the level of induced expression in Ziyu44 is much lower than in JNXN. miR9664 targets *R* genes greatly induced on blast infection in rice, like *LOC_Os08g07774, LOC_Os08g42700*, and *LOC_Os12g13550*. In Ziyu44, low miR9664 levels result in the accumulation of H_2_O_2_ and enhanced resistance to *M. oryzae*. In susceptible rice varieties, like JNXN, miR9664 was sharply induced, there was an accumulation of high-level miR9664, the levels of its target genes greatly decreased, specific H_2_O_2_ degradation activities were activated, leading to susceptibility.

Despite many reports on the involvement of miRNAs in rice immunity against rice blast fungus, there are few reports on miRNAs regulating the broad-spectrum and durable blast resistance in rice. Our results show that a higher quantity of significantly differentially expressed miRNAs are identified in Ziyu44 than in JNXN after being infected with rice blast fungus (Figure 2), suggesting that miRNAs play important roles in the regulation of broad-spectrum and durable blast resistance in the *japonica* rice variety, Ziyu44. miR9664, a novel rice miRNA identified in our current study, is upregulated lightly in Ziyu44 and drastically in JNXN, especially at 24–48 hpi, and mediated *R* gene turnover contributes to the broad-spectrum resistance to rice blast fungus of Ziyu44. We suggest that the slight upregulation of miR9664 is conducive to disease resistance in rice, while a sharp up-regulation will lead to susceptibility to rice blast, and a model is presented to summarize our hypothesis (Figure 9).

In our study, although a panel of differently expressed miRNAs was identified in the Ziyu44 and JNXN infected with *M. oryzae*, we found that the number and types of differentially expressed miRNAs have a large variation among biological replications (Figure 2A, Supplementary Table 2). We think this situation is most likely caused by the design of the biological replicates. In our study, three biological repeats were completed in three batches, including rice seedling cultivation and preparation of spore suspension. The time interval between the biological replicates is about 10 days and some uncontrollable environmental factors may lead to certain differences in spore vitality and rice seedlings' growth. To ensure the reliability of the analysis results, our analysis mainly focused on the constantly differentially expressed miRNAs in the three biological replicates.

Most of the reported involvement of miRNAs in rice immunity against rice blast fungus is identified in our study (Table 1). In addition to the known miRNAs involved in rice immunity against *M. oryzae*, some miRNAs. differentially expressed in the Ziyu44 infected with rice blast fungus, have not been reported to be involved in the rice response to *M. oryzae*, such as osa-miR1856, osa-miR1862d, osa-miR3980a-5p, miR9664, etc. (Table 1), which may play important roles in the regulation of broad-spectrum and durable blast resistance in Ziyu44. Besides, increasing evidence showed that plant *R* genes are under direct targeting by miRNAs (Ouyang et al., 2014; Baldrich and San Segundo, 2016; Deng et al., 2018; Prigigallo et al., 2019; Zhang R. et al., 2019; Zhang Y. et al., 2019; Cui et al., 2020). However, until now, only osa-miR2055, osa-miR2864.2, osa-miR2870 (Hong et al., 2015), and osa-pm5124 (Lian et al., 2016), four rice miRNAs (osa-miRNAs) targeting *R* genes are reported. miR9664-mediated *R* genes turnover contributes to the broad-spectrum resistance of Ziyu44 against rice blast fungus: a slight upregulation of miR9664 is conducive to disease resistance in Ziyu44 whereas a sharp up-regulation will lead to rice susceptibility. However, we found that only three (*LOC_Os08g42700, LOC_Os08g07774*, and *LOC_Os12g13550*) out of the 19 predicted target genes examined in our study were decreased in two miR9664-oe lines and increased in two miR9664-m lines (Figures 5B, 6B, Supplementary Figures 5, 6), and the specific cleavage within the miR9664 target site was only confirmed in *LOC_Os08g07774* (Figure 8B, Supplementary Figure 7). In addition, upon being infected with *M. oryzae*, the expression of the 19 predicted target genes of miR9664 was largely varied in the resistant cultivar Ziyu44 and susceptible cultivar JNXN (Figure 8A). Therefore, the studies on how miR9664 differentially regulates the expression of different target genes, how the expression levels of miR9664 and its target genes are coordinated during Ziyu44–*M. oryzae* interactions, the targeting relationship of other genes with miR9664, and the functional analysis of target genes will help us understand the molecular mechanism of miR9664 in regulating disease resistance against *M. oryzae* in Ziyu44 better.

The identification of the broad-spectrum blast resistance genes and study of the molecular basis of broad-spectrum resistance are critical to ensure global food security. However, most of the identified broad-spectrum blast resistance resources are *indica* rice varieties, such as Digu and Gumei4 (Deng et al., 2017; Li W. et al., 2017), the research of the broad-spectrum blast resistance mechanism is also limited to *indica* rice and the resistance genes cloned from these materials. Yunnan is the main rice-growing area in southern China. Rice varieties and cultivation systems are diverse. Ziyu44, a local *japonica* rice variety, was bred from a cross between the Yunnan local varieties, Mazaogu and Chengbao2, in 1987 (Zhang et al., 2009). Over the past 30 years, Ziyu44 has displayed high resistance to rice blasts in fields. In our previous study, multiple major resistance genes and quantitative trait loci (QTLs) were identified in Ziyu44 (Zhang et al., 2011; Zhou et al., 2015; Zhuo et al., 2019). In this study, we identified multiple miRNAs involved in Ziyu44 immunity against rice blast. Therefore, we conclude that the mechanism of the broad-spectrum blast resistance of Ziyu 44 is complex. More systematic and in-depth studies are still needed to know how major genes, minor genes, and miRNAs coordinate the regulation of rice blast resistance in Ziyu44.

## Data Availability Statement

The datasets presented in this study can be found in online repositories. The names of the repository/repositories and accession number(s) can be found at: NCBI (accession: PRJNA756698).

## Author Contributions

QL was responsible for the research design, funding acquisition, project administration, and for the interpretation of results. JL conducted the experiment and was responsible for the analysis and interpretation of data. HZ performed the isolation and preservation of the M. oryzae strains. RY prepared the leaves samples for sRNA-Seq and mRNA-Seq. QL and JL wrote and edited the manuscript. QZ provided the rice varieties. GH supported the field experiments. JY performed the microscope related experiments. YD and GY helped draft the manuscript. All authors read and approved the final manuscript.

## Funding

This work was supported by grants from the National Science Foundation of China (31960568 and 31160223), the National Key Research Development Program of China (2016YFD0100600), Yunnan Agricultural University Outstanding Scholar Project (2020JY04), Yunnan Agricultural University ESI Discipline Promotion Program (2019YNAUESIMS02), and the Program for Innovative Research Team in University of Yunnan Province (IRTSTYN).

## Conflict of Interest

The authors declare that the research was conducted in the absence of any commercial or financial relationships that could be construed as a potential conflict of interest.

## Publisher's Note

All claims expressed in this article are solely those of the authors and do not necessarily represent those of their affiliated organizations, or those of the publisher, the editors and the reviewers. Any product that may be evaluated in this article, or claim that may be made by its manufacturer, is not guaranteed or endorsed by the publisher.
