# Supplementary material for: Identification of miRNAs Contributing to the Broad-Spectrum and Durable Blast Resistance in the Yunnan Local Rice Germplasm
Source: Front Plant Sci. 2021 Oct 14;12:749919. doi: 10.3389/fpls.2021.749919 (PMC8551726; doi:10.3389/fpls.2021.749919)
Supplement: Supplementary file 6 [file Data_Sheet_1.docx]

# SUPPORTING INFORMASTION


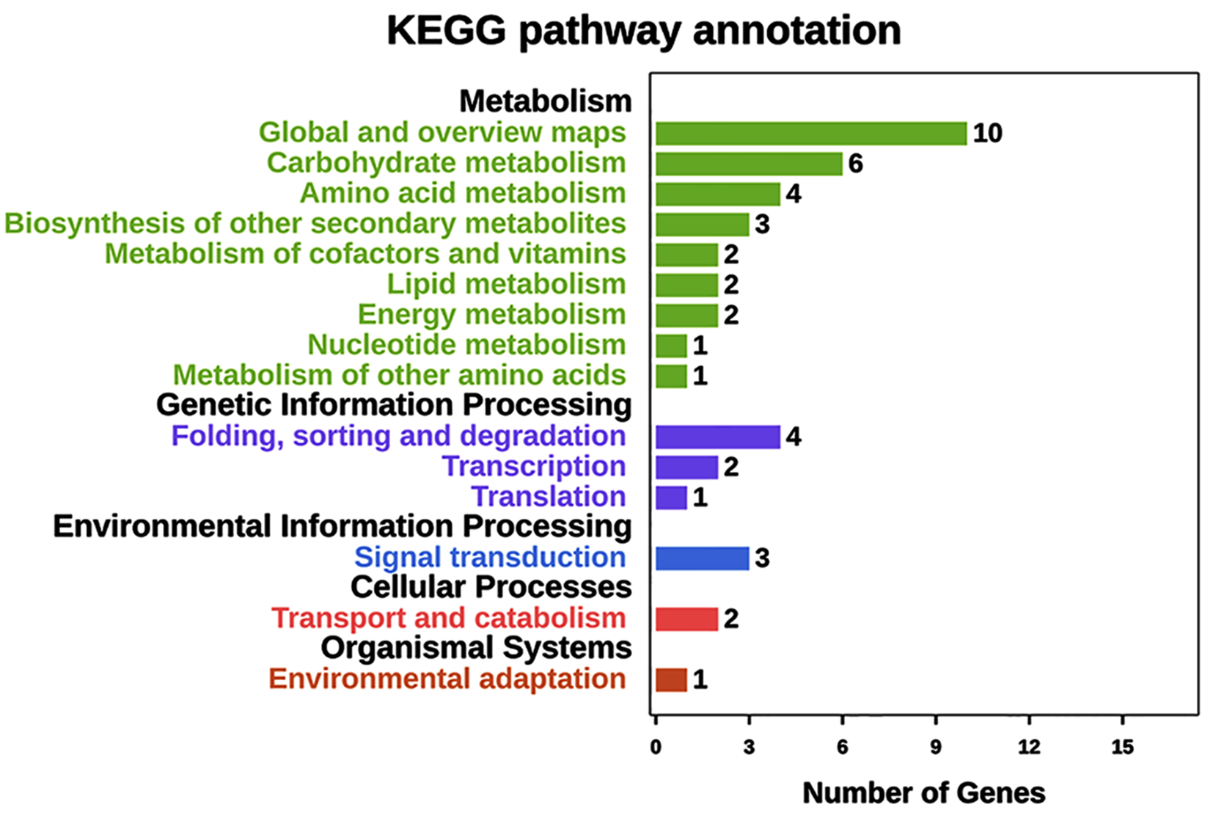


**Figure S1** The top 20 Kyoto Encyclopedia of Genes and Genomes (KEGG) pathway terms. This graph counts the number of genes annotated to pathway A and B. The ordinate in the figure is KEGG’s A-level and B-level categories. The black font is the A-level category name, and the color font is the B-level category name. The abscissa is the number of genes in the corresponding B-level classification.

**
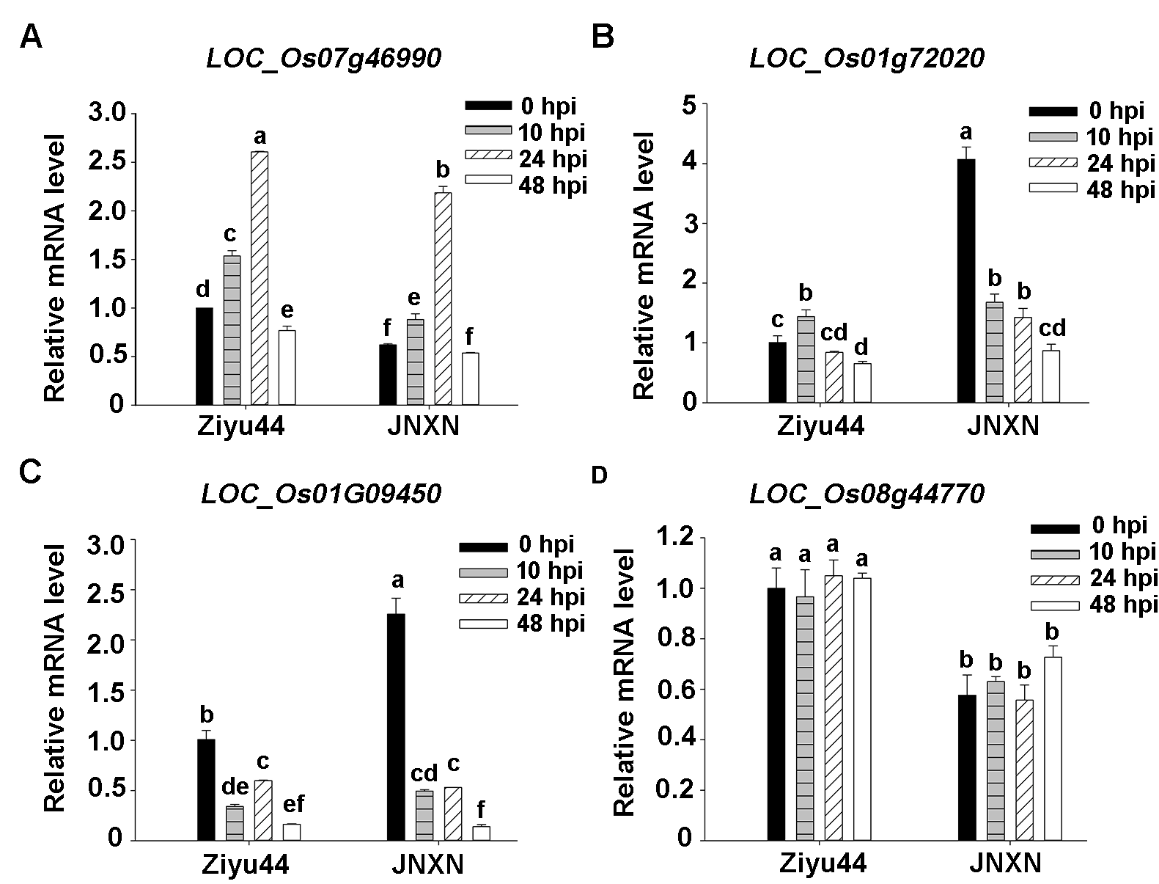
**

**Figure S2** RT-qPCR verification of gene expression in Table2. RT-qPCR data show the relative messenger RNA (mRNA) of *LOC_Os07g46990* (A), *LOC_Os01g72020* (B), *LOC_Os01g09450* (C) and *LOC_Os08g44770* (D) in Ziyu44 and JNXN at the indicated time points upon inoculation of conidial mixture of 4 *M. oryzae* strains (81278, ZB13, TC61 and H63) . RNA was extracted at the indicated time points. The indicated mRNA level is normalized to that in the untreated control plants (0 hpi). Error bars indicate SD (n=3). The letters above the bars indicate significant differences at a value of P＜0.01 as determined by One-way analysis of variance (ANOVA) followed by *post hoc* Tukey HSD analysis.


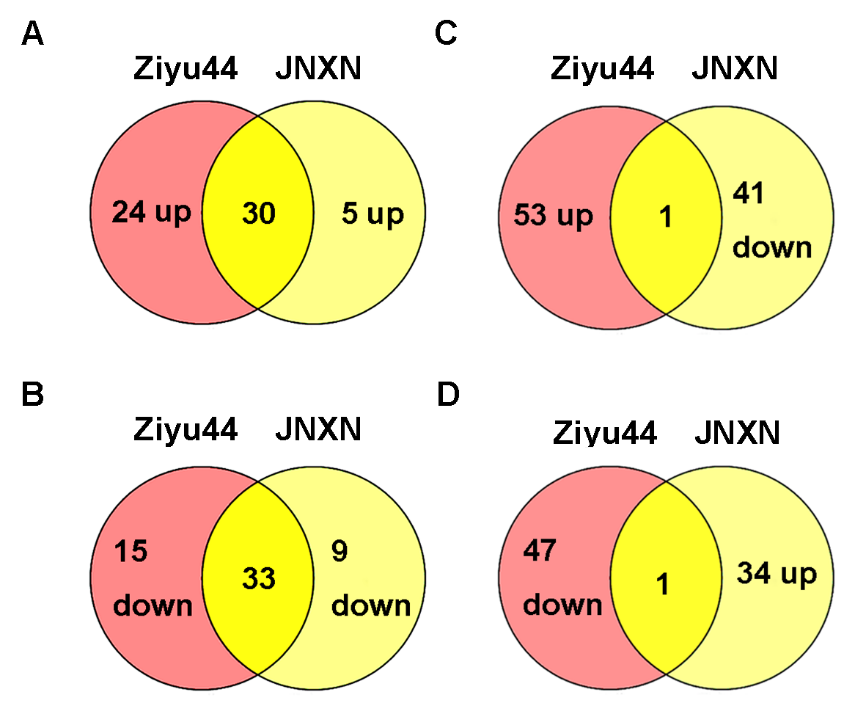


**Figure S3** Venn diagram showing the distribution of predicted target genes which differentially expressed in Ziyu44 and JNXN.


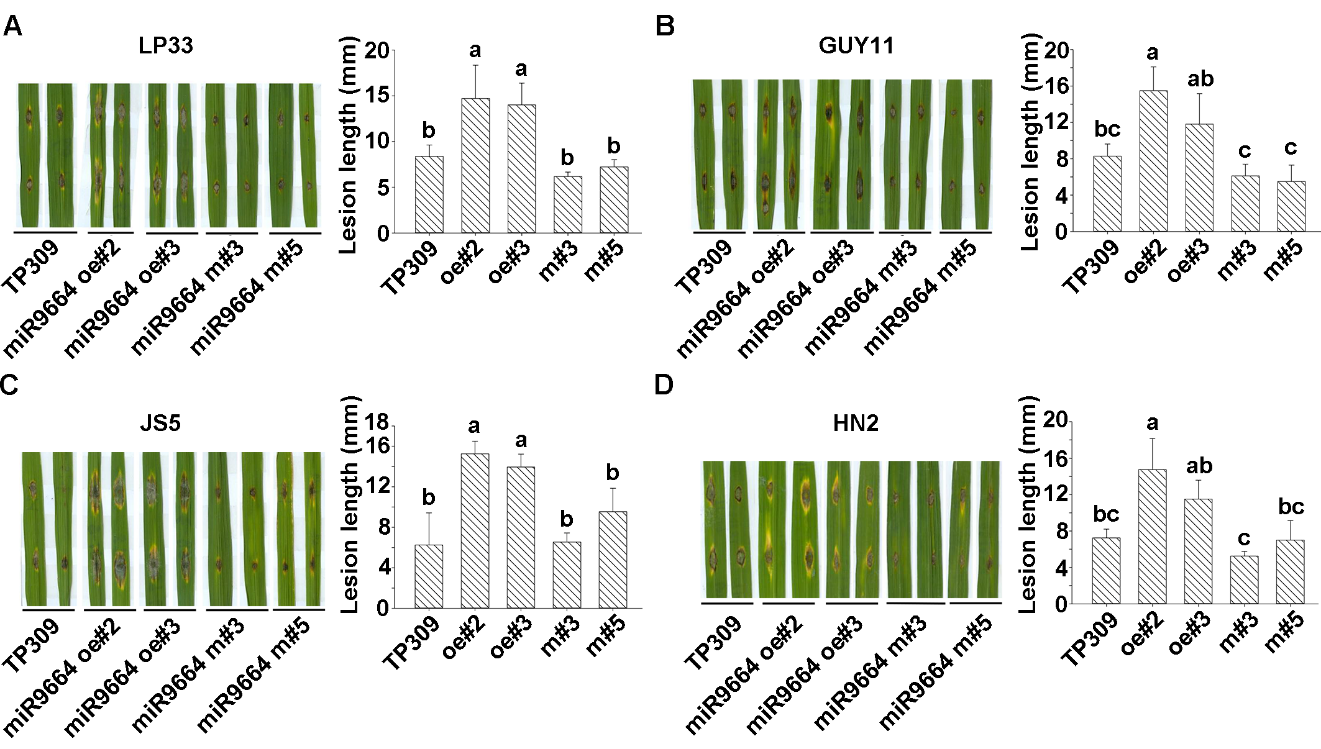


**Figure S4** Blast Resistance of TP309, miR9664 oe#2, miR9664 oe#3, miR9664 m#3 and miR9664 m#5 plants using punching inoculation with *M. oryzae* strain LP33 (A), GUY11(B), JS5(C) and HN2 (D). Two inoculated leaves each of are shown. Lesion length was measured by Caliper. Error bars indicate SD (n=12), the letters above the bars indicate significant differences (P＜0.01).


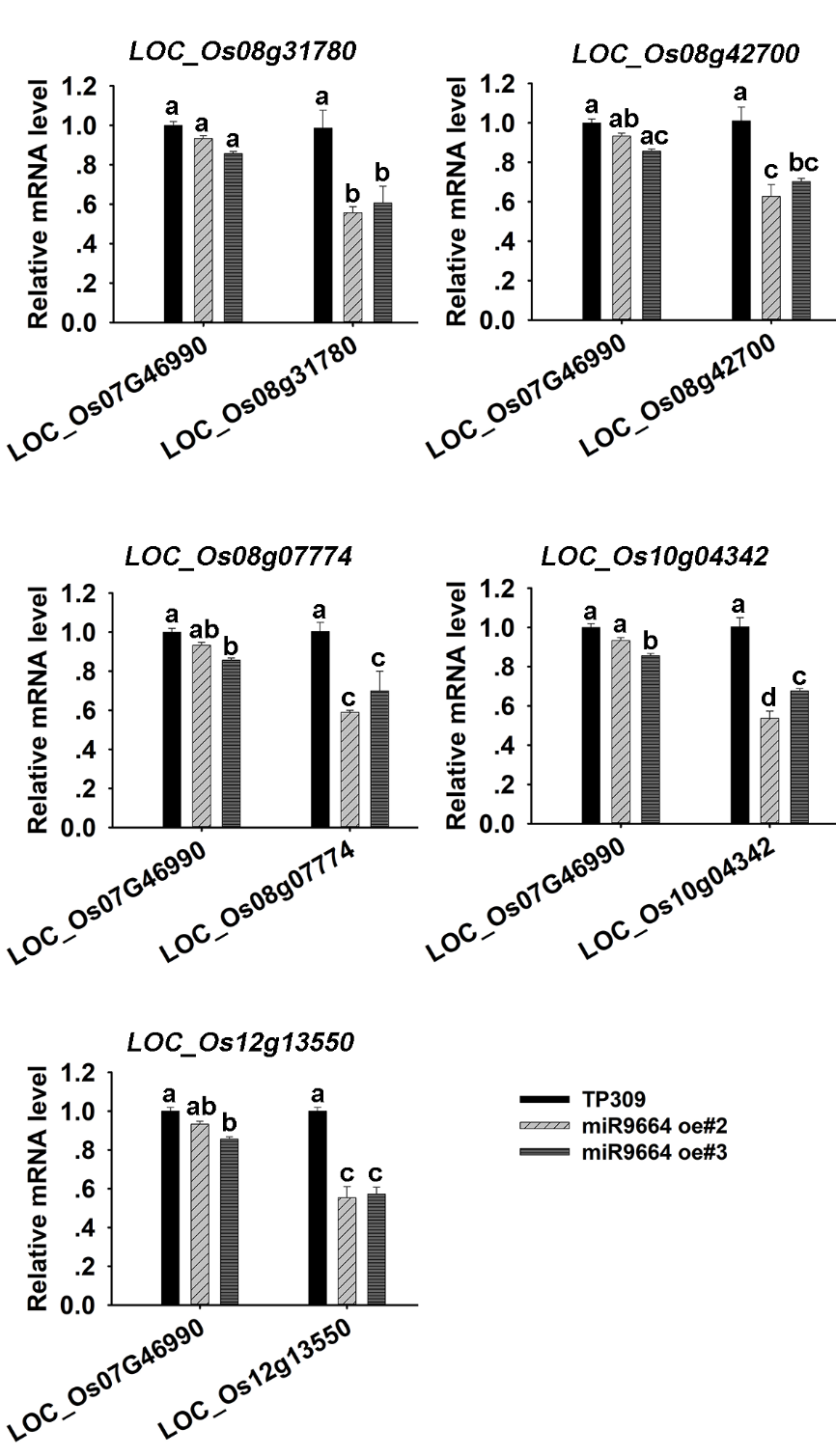


**Figure S5** The expression levels of *LOC_Os08g31780*, *LOC_Os08g42700*, *LOC_Os08g307774*, *LOC_Os10g04342* and *LOC_Os12g13550* of miR9664 predicted target genes were significantly down-regulated in both two miR9664-oe lines compared to a non-target gene *LOC_Os07g46990*.


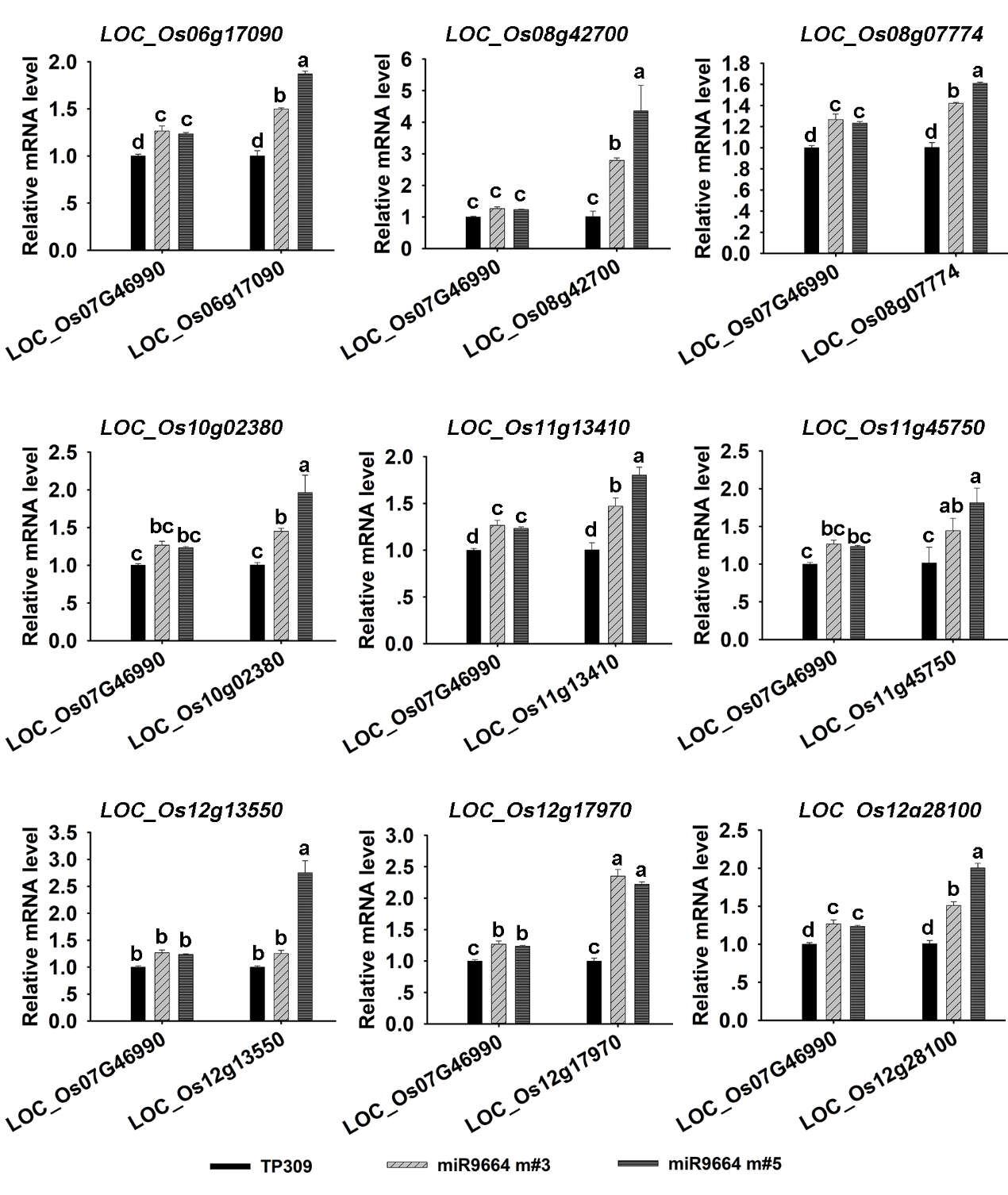


**Figure S6** The expression levels of *LOC_Os06g17090, LOC_Os08g42700, LOC_Os08g07774, LOC_Os10g02380, LOC_Os11g13410, LOC_Os11g45750, LOC_Os12g13550, LOC_Os12g17970* and *LOC_Os12g28100* of miR9664 predictaed target genes were significantly up-regulated in both two miR9664-m lines compared to a non-target gene *LOC_Os07g46990*.


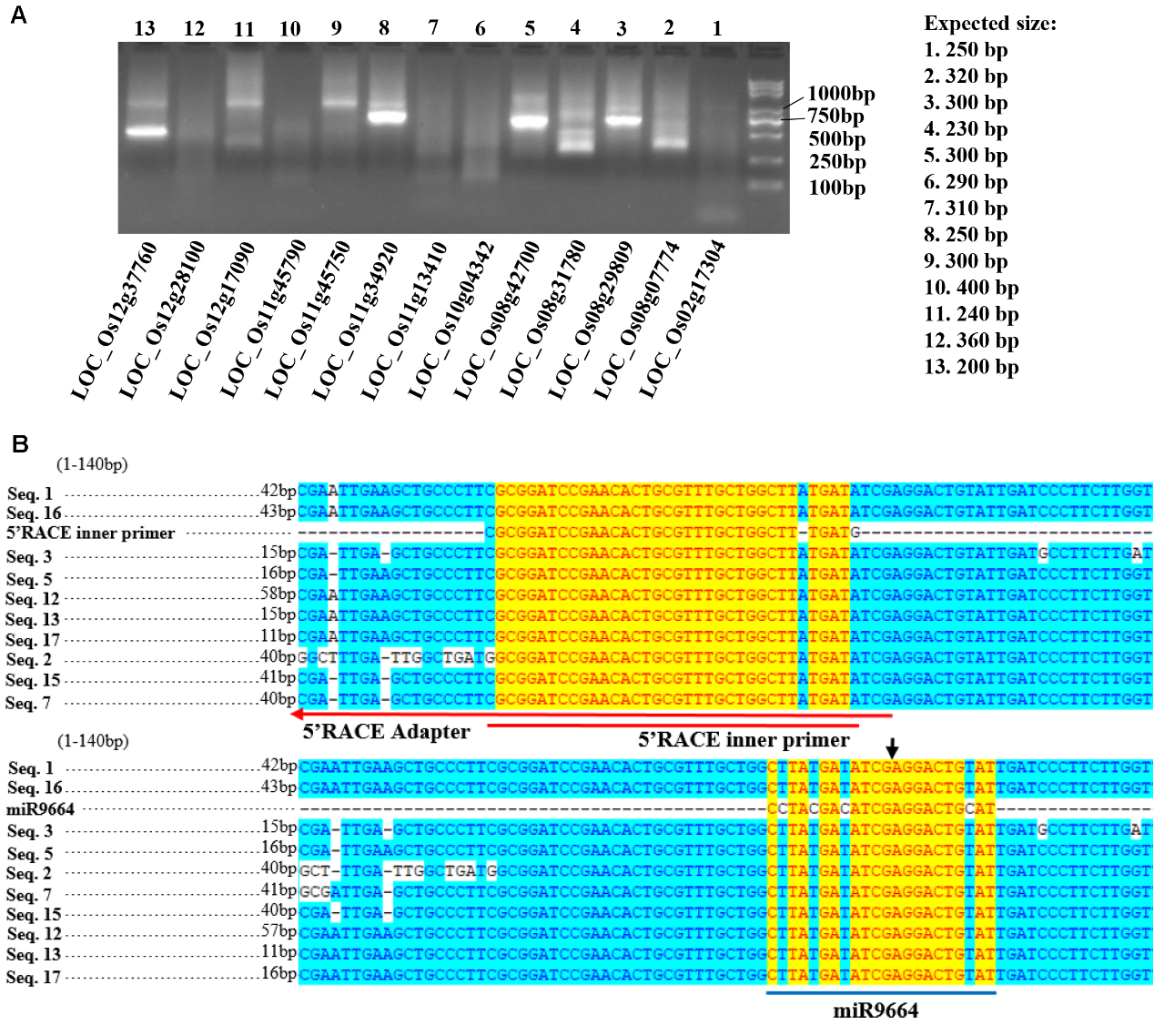


**Figure S7**. Results of 5’RLM-RACE for 13 predicted target genes of miR9664. (A) PCR result amplified with inner primer. (B) Sequencing result of *LOC_*Os08g07774. The sequence of 5’RACE adapter is underlined in red, 5’RACE inner primer sequence is underlined in double red. The sequence of miR9664 is underlined in blue. 5’RACE adapter contains 5’RACE inner primer. The black arrow indicated the cleavage site.

Table S1 Distribution of sRNAs annotation in each library

Table S2 Significantly differentially expressed miRNAs

Table S3 Target prediction of significantly differentially expressed miRNAs

Table S4 Significantly differentially expressed predicted target genes of miRNAs

Table S5 Primers used in this study
